# Biomarkers predicting adverse pregnancy outcomes in women living with obesity: a systematic review and meta-analysis

**DOI:** 10.1016/j.xagr.2025.100527

**Published:** 2025-07-22

**Authors:** Tabitha Wishlade, Sara Wetzler, Catherine E. Aiken

**Affiliations:** 1 Department of Obstetrics and Gynaecology, The Rosie Hospital and NIHR Cambridge Biomedical Research Centre, University of Cambridge, Cambridge, United Kingdom (Wishlade and Aiken); 2 Department of History and Philosophy of Science, University of Cambridge, Cambridge, United Kingdom (Wetzler); 3 Department of Obstetrics, Gynecology, and Reproductive Science, Icahn School of Medicine at Mount Sinai, New York, NY (Wetzler)

**Keywords:** adiponectin, biomarkers, gestational diabetes, meta-analysis, obesity, pregnancy outcomes, pre-eclampsia

## Abstract

**Objective:**

To systematically review the literature on associations between antenatal biomarkers and adverse pregnancy outcomes in women with BMI ≥30 kg/m². *Data sources*: Systematic literature searches used predefined search terms in PubMed, Ovid Embase, Ovid MEDLINE, Scopus, and the Cochrane Central Register of Controlled Trials. Databases were searched from inception to August 2024.

**Study eligibility criteria:**

Interventional and observational studies comparing pregnancy outcomes amongst women with a pre- or early-pregnancy (<20 weeks’ gestation) BMI ≥30 kg/m² according to presence or amount of any antenatally measured biomarker were included.

**Study appraisal and synthesis methods:**

Two reviewers independently assessed studies for inclusion against predefined inclusion and exclusion criteria. Risk of bias assessment was performed on included studies using the Newcastle-Ottawa Risk of Bias Tool. A narrative synthesis of eligible studies was constructed and data were meta-analysed, where possible, using random effects models. Certainty of evidence was assessed by GRADE rating.

**Results:**

Total 49 studies were included in the review, representing >500 different biomarkers in 7479 pregnancies across 16 countries. Adiponectin was the only biomarker with sufficient data to meta-analyse with respect to the composite outcome. Lower adiponectin was associated with increased risk of the composite outcome (SMD −0.52, 95% CI −0.63, −0.41; *P*<.001, I^2^ 0%). Lower adiponectin was also associated with increased risk of gestational diabetes (SMD −0.57, 95% CI −0.70, −0.43; *P*<.001, I^2^ 0%) and pre-eclampsia (OR 0.65, 95% CI 0.44, 0.99; *P*=.047, I^2^ 61.5%). Increased insulin concentrations were associated with gestational diabetes (SMD 0.35, 95% CI 0.22, −0.47; *P*<.001, I^2^ 0%). Certainty of evidence regarding all associations was low or very low.

**Conclusions:**

Decreased adiponectin and increased insulin are associated with increased risk of adverse pregnancy outcomes in women with BMI ≥30 kg/m². However, the low number of studies available for inclusion and low certainty of evidence mean that biomarker-based risk-stratification within pregnant women with BMI ≥30 kg/m² is not currently feasible. Further research is required to find ways of reliably targeting investigations during maternity care towards the subset of women living with obesity who are at highest risk of adverse outcomes.

**AJOG Global Reports at a Glance**

**Why was this study conducted?**

Pregnant women living with obesity are at higher risk of adverse pregnancy outcomes than their lean counterparts, yet the majority of women with body mass index (BMI) ≥30 kg/m² will have a normal pregnancy. There is a need for improved risk stratification for pregnant women with BMI ≥30 kg/m². Antenatal maternal biomarkers may help identify women with BMI ≥30 kg/m² who are at increased risk of adverse pregnancy outcomes.

**Key findings**

Few studies report biomarkers in populations of women with BMI ≥30 kg/m² leading to low certainty of evidence. Maternal circulating adiponectin and insulin concentrations may be associated with increased risk of adverse pregnancy outcomes in women with BMI ≥30 kg/m².

**What does this add to what is known?**

More research is needed to support accurate risk stratification for the increasing population of pregnant women living with obesity

Further evidence is needed to refine the potential use of adiponectin or insulin as a biomarker in women with BMI ≥30 kg/m²

## Introduction

16% of the world’s current pregnant population lives with obesity,^1^ but with obesity expected to affect 24% of the world’s population by 2035,^2^ this number is set to rise. In the UK, 18% of primiparous women and 25% of multiparous women commence pregnancy with BMI ≥30 kg/m².^3^ Maternal obesity is associated with later-life maternal cardiometabolic ill-health^4^, ^5^, ^6^ and increased offspring adiposity and cardiometabolic risk.^7^^,^^8^ These women are also at higher risk of adverse pregnancy outcomes, including gestational diabetes (GDM),^9^ preeclampsia,^10^ and preterm delivery^11^ which are major causes of maternal and infant morbidity and mortality globally.^12^^,^^13^ Indeed, women with obesity are more at risk of maternal mortality than those who are not.^14^

In recognition of this increased risk, antenatal care pathways for pregnant women with BMI ≥30 kg/m² usually include increased surveillance, testing, and recommendations for interventions.^11^^,^^15^^,^^16^ However, most pregnant women with obesity will have an uncomplicated pregnancy.^17^ Studies suggest ∼90% do not develop preeclampsia,^18^^,^^19^ >80% do not develop GDM^20^, ^21^, ^22^ and 85% do not have macrosomic infants.^23^^,^^24^

As obesity rates in women of child-bearing age rise globally, including in low resource populations, it becomes increasingly expensive and burdensome to offer significant additional antenatal care to all women living with BMI ≥30 kg/m². Moreover, clinicians may cause unintended psychological harm from increased anxiety and stigmatisation by offering additional testing and interventions to women living with obesity who might otherwise have a healthy pregnancy.^25^, ^26^, ^27^ A more nuanced approach to risk stratification than using BMI alone is therefore needed to allow the lowest-risk pregnant women living with obesity to avoid unnecessary interventions whilst enabling those at highest risk to receive timely specialist care.

The circulating metabolic milieu of pregnant women living with obesity differs from that of women with BMI <30 kg/m² even when conditions such as gestational diabetes and hypertension are controlled for.^28^^,^^29^ Thus, the identification of biomarkers associated with increased risk of adverse outcomes specific to pregnancies affected by obesity may be a method of refining risk-stratification. Biomarkers - either causal or associative - could help to refine individual assessments of risk, ensure efficient allocation of resources, and potentially contribute to improved understanding of the mechanisms underlying the increased risk of adverse outcomes.

Over the past decade more studies have emerged suggesting that biomarker concentrations, for example low concentrations of PAPP-A^30^ and high HbA1c,^31^ are associated with poor pregnancy outcomes in the context of maternal obesity.^32^^,^^33^ However, heterogeneity in both outcomes and exposure makes comparisons across studies complex. We therefore aim to conduct an exploratory systematic review and meta-analysis of existing evidence on the associations between antenatal biomarkers and adverse pregnancy outcomes in women with BMI ≥30 kg/m^2^.

## Objective

We undertook a systematic review and meta-analysis of published literature on associations between antenatal biomarkers and adverse pregnancy outcomes in pregnant women living with obesity.

## Material and methods

This systematic review and meta-analysis was reported in accordance with the Preferred Reports for Systematic Reviews and Protocol Meta-Analysis (PRISMA-P)^34^ (Supplementary Table 1), and was prospectively registered with the international prospective register of systematic reviews (PROSPERO; CRD42023487129).

### Eligibility criteria, data sources, and search strategy

We defined “living with obesity” as BMI ≥30 kg/m^2^ either pre- or early-pregnancy (<20 wks gestation). BMI was either self-reported or measured as part of routine clinical care or during study procedures. Although self-reported BMI may lead to under-estimation of weight and thus BMI in women^35^ leading to misclassification bias, it has been shown women correctly self-classify as obese in 92% of cases.^36^

Biomarkers were defined as any marker (either quantitative or qualitative) feasibly measured in biological samples obtained from the mother (blood, urine, etc.) at any stage during pregnancy. We excluded markers that could only feasibly be assayed at delivery or postnatally, for example in samples from cord blood or placental tissue. We further excluded imaging findings as biomarkers. Adverse outcomes were defined as per the original studies; the definitions used for each study are listed in Supplementary Table 2.

Interventional and observational studies were considered for inclusion. Studies were included if they reported (1) research in humans using prospective or retrospective design and (2) comparison of pregnancy outcomes amongst women with BMI ≥30 kg/m² according to presence or amount of any antenatally measured biomarker. Studies were excluded if they were (1) animal studies, (2) systematic reviews/meta-analyses, or (3) had fewer than 20 relevant participants. Conference abstracts/proceedings were also excluded. No language, date, or geographical restrictions were applied.

To identify eligible studies, systematic literature searches were performed using predefined search terms (Appendix A) developed in liaison with an information specialist. Searches were conducted in PubMed, Ovid Embase, Ovid MEDLINE, Scopus, and the Cochrane Central Register of Controlled Trials (CENTRAL). Databases were searched from inception until August 19, 2024. Duplicate studies were excluded using EndNote v21 and Rayyan (https://rayyan.ai) software.

The full text of one article^37^ could not be retrieved and the authors could not be contacted. Three further study authors were contacted to clarify points of information.^38^, ^39^, ^40^ Two authors responded with further information. One author had retired and had no access to the data.

### Study selection

Two reviewers (TW and SW) independently performed (1) abstract and title screening followed by (2) full text screening according to the prespecified inclusion and exclusion criteria. Any differences in opinion were resolved by a third reviewer (CEA). Data were extracted by one reviewer and independently validated by another reviewer.

### Data extraction

Data were extracted into a prespecified format. Variables for which data was extracted are listed in Appendix B. Data was not estimated from figures, but study authors were contacted to provide clarifications where required. Two study authors were contacted at this stage to aid data extraction.^41^^,^^42^ Both authors provided the requested data. The study outcomes were prespecified in the PROSPERO registration.

### Assessment of risk of bias

Risk of bias assessment was carried out on all included studies using the Newcastle-Ottawa Risk of Bias Tool.^43^ Assessment was performed by one reviewer and verified independently by a second reviewer, with a third reviewer available for disagreements.

### Data synthesis

An *a priori* decision to use a random effects model was made in view of the anticipated clinical heterogeneity in studies. Where data were presented as means and standard deviations, standardized mean difference (SMD) was used as the effect measure for continuous data to account for biomarkers being measured in different units. Hedge’s *g* was used to calculate the SMD due to its correction to Cohen’s *d* for small sample bias.^44^ Where median and IQR were presented for continuous variables, difference in medians was calculated. The odds ratio ±95% confidence interval was used as the measure of effect for dichotomous data. Where the same biomarker was measured in different units across studies and standardized mean difference could not be calculated, biomarker data were transformed into a common unit of measurement, thereby enabling the difference in medians to be calculated. Biomarker data were presented on nonlinear scales in several studies. For standardized mean differences, mean and standard deviation data were transformed to the natural log scale before pooling, according to published methods.^45^^,^^46^ For odds ratios, log₂ data was removed in sensitivity analyses when other odds ratios were derived from natural log data.

Meta-analysis was performed when there were at least two studies reporting the same biomarker for the same outcome. Where biomarker data were presented for sub-groups, we combined these where possible. If it was not possible to combine groups, we selected the subgroup that minimized heterogeneity across the included studies in each meta-analysis.

A composite measure of "poor pregnancy outcome," combining biomarker data across outcomes, was used. Meta-analysis was performed when there was data on one biomarker across at least two different outcomes.

*I*^2^ and *Q* statistics were used to assess study heterogeneity. Leave-one-out analysis was performed on all meta-analyses that included three or more studies. Funnel plots with trim-and-fill were constructed for all biomarker-outcome associations with ≥3 studies. Egger’s regression test was performed on all meta-analyses containing 4 or more studies. GRADE assessment was performed to assess the quality of evidence.

Meta-analysis was performed using the *meta* (7.0-0) and *metafor* (4.6-0) and *metamedian* (1.1.1) packages in R (version 4.3.3). When it was not possible to combine data from eligible studies in meta-analysis, a narrative synthesis was constructed.

## Results

### Study selection

**Figure 1:**
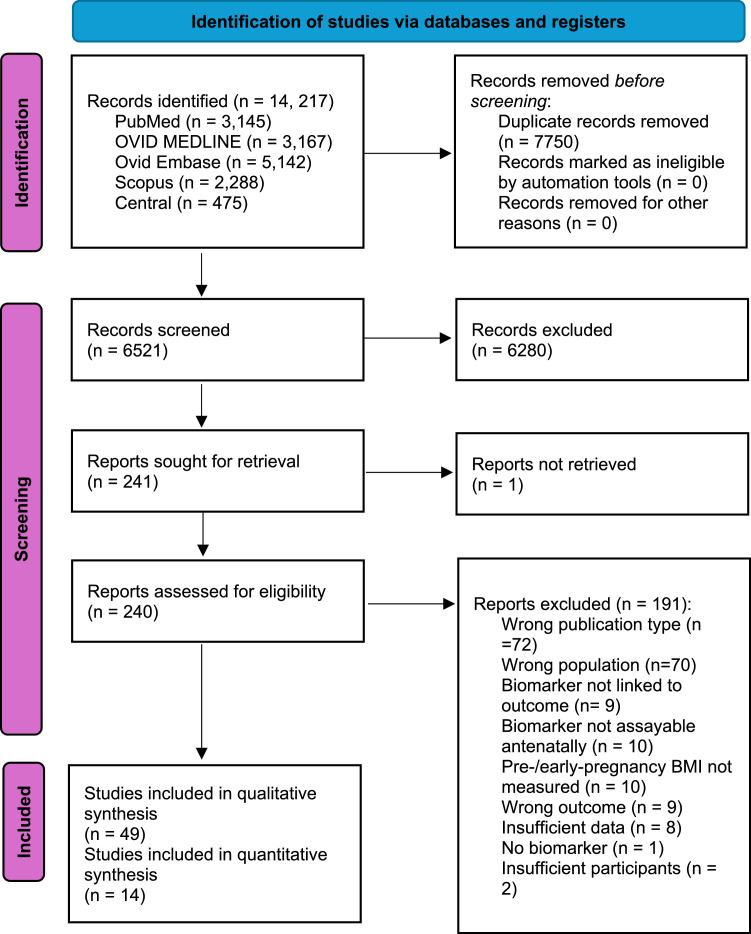
Flow diagram illustrating the identification and screening phases of the systematic review

Our searches identified 14,619 records, of which 49 studies met the inclusion criteria (Figure 1) representing >500 different biomarkers in 7479 pregnancies across 16 countries.

Wishlade et al. Biomarkers predicting adverse pregnancy outcomes in women living with obesity. Am J Obstet Gynecol 2025.

### Risk of bias of included studies and sensitivity analysis

Studies were assessed as having low (n=29), medium (n=14), or high (n=6) risk of bias (Table). Sensitivity analysis excluding all studies with a low or moderate risk of methodological bias (quality scores <7) was performed, with the majority (5 out of 7) meta-analyses robust to excluding these studies (Supplementary Table 3). Several meta-analyses (5 out of 21) were not robust to leave-one-out analysis, reducing our confidence in these findings (Supplementary Table 3). Due to a limited number of studies in most meta-analyses, funnel plot asymmetry could not be assessed (Supplementary Figure 1). Trim-and-fill methods did not appreciably change any effect estimates (Supplementary Figure 1). Egger’s test was not significant for any meta-analysis where it was performed (Supplementary Table 3). GRADE assessment was performed on all meta-analyses; the body of evidence included in this review was low or very low (Supplementary Table 4).

**Table:** Table illustrating the risk of bias in each of the included studies across different domains

Green circle = low risk of bias; yellow circle = unknown risk of bias; red circle = high risk of bias.

### Synthesis of results

#### Composite poor pregnancy outcome

**Figure 2:**
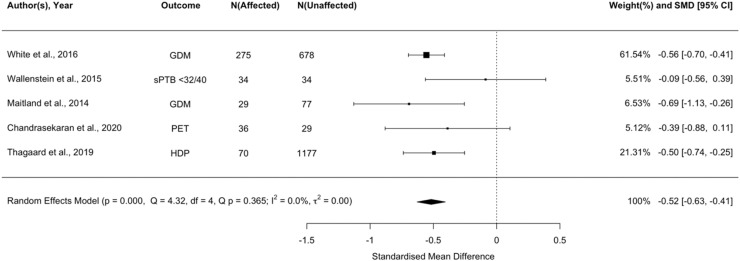
Forest plot of standardised mean difference in log adiponectin between pregnant women with a BMI ≥ 30 kg/m^2^ who experience an adverse pregnancy outcome vs those who do not GDM = gestational diabetes; sPTB <32/40 = spontaneous pre-term birth under 32 weeks’ gestation; PET = pre-eclampsia; HDP = hypertensive disorders of pregnancy.

We examined whether any biomarker was associated with a prespecified composite measure of any adverse pregnancy outcome in women living with obesity. Adiponectin was the only biomarker with sufficient data to meta-analyse with respect to the composite outcome. Lower adiponectin was associated with increased risk of any adverse pregnancy outcome (Figure 2; *P*<.001). However, GRADE-assessed certainty of evidence was low.

Wishlade et al. Biomarkers predicting adverse pregnancy outcomes in women living with obesity. Am J Obstet Gynecol 2025.

#### Gestational diabetes mellitus (GDM)

GDM was reported across 28 studies including 4135 participants. Of the included studies, 11 were secondary analyses of data from a single randomised controlled trial, the UPBEAT study.^47^ Sensitivity analysis was performed excluding studies derived from UPBEAT for each of the 10 meta-analyses relating to biomarkers of GDM. Exclusion of the UPBEAT studies did not materially alter the estimate of effect in any case, but reduced power, causing 3 out of 4 meta-analyses with *P* values for effect <.05 to become >.05 (Supplementary Table 5).

### Insulin

**Figure 3:**
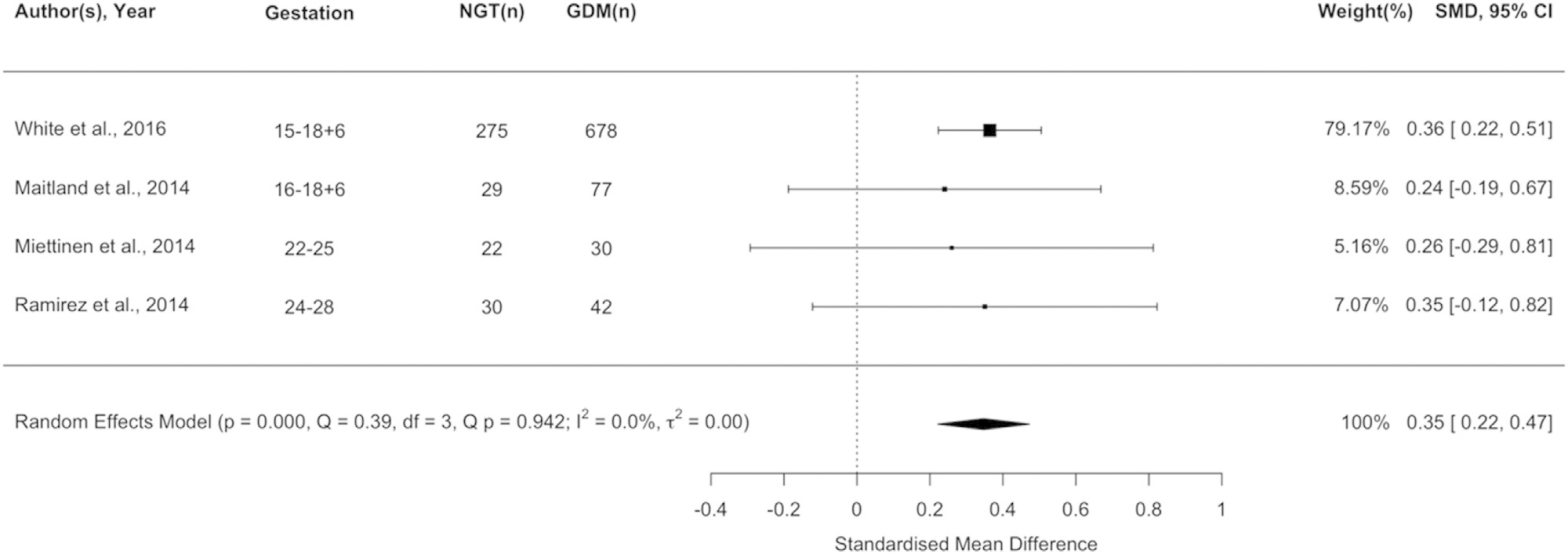
Forest plot of standardised mean difference in log insulin between pregnant women with a BMI ≥ 30 kg/m^2^ who experience gestational diabetes and those remaining glucose tolerant

Eleven studies (n=1802) reported insulin as a possible biomarker for GDM. Higher insulin was associated with a greater risk of GDM v. normal glucose tolerance (NGT) (*P*<.001; Figure 3). To assess the strength of the association between elevated insulin levels and GDM at different gestational ages we performed a meta-regression (<20 weeks v. >20 weeks; Supplementary Figure 2). Gestational age at sampling did not affect the relationship between increased insulin and likelihood of developing GDM in women with BMI ≥30 kg/m².

Wishlade et al. Biomarkers predicting adverse pregnancy outcomes in women living with obesity. Am J Obstet Gynecol 2025.

#### Narrative synthesis of additional studies

Elevated circulating insulin concentrations were found in women who experienced GDM women v. those with NGT up to 10 weeks before diagnosis in 3 additional cohort studies (n=867,^48^ n=646,^49^ n=111,^50^ respectively), 2 of which were derived from the UPBEAT trial. Both a high-risk sub-group from UPBEAT (n=231)^51^ and a case control study (n=82)^52^ also showed a positive correlation between insulin and GDM risk but not to the level of statistical significance. Two further studies (n=300,^53^ n=867^54^ respectively) drawn from the UPBEAT found insulin concentrations may vary by GDM subtype in women with BMI ≥30 kg/m².

There is thus some evidence that higher insulin concentrations are associated with an increased risk of GDM among women with BMI ≥30 kg/m², even when measured early in pregnancy. However, the GRADE certainty assessment of meta-analyzed evidence was very low.

### Adiponectin

**Figure 4:**
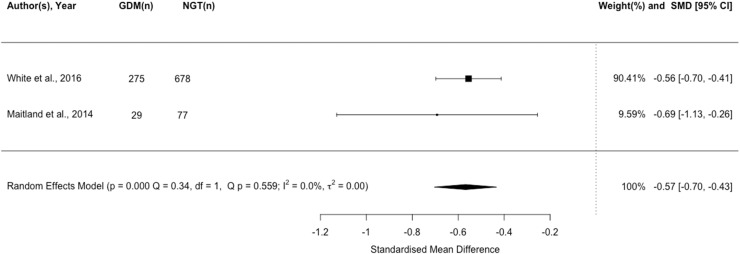
Forest plot of standardised mean difference in log adiponectin between pregnant women with a BMI ≥ 30 kg/m^2^ who experience gestational diabetes and those remaining glucose tolerant

Seven studies (n=2805) reported associations between adiponectin and the risk of GDM. Lower adiponectin concentrations were associated with an increased risk of GDM v. NGT (*P*<.001; Figure 4).

Wishlade et al. Biomarkers predicting adverse pregnancy outcomes in women living with obesity. Am J Obstet Gynecol 2025.

*Narrative synthesis of additional studies:* One large retrospective (n=1292^55^) and three smaller prospective cohort studies (n = 646,^49^ n=231,^51^ and n=72,^56^ respectively) all suggest lower circulating adiponectin concentrations are associated with developing GDM v. NGT. The association was robust to timing of adiponectin measurement, including up to 14 weeks prior to diagnosis. However, one small case control study (n=21^57^) found no difference in adiponectin levels in GDM v. NGT prior to elective caesarean section.

Available evidence suggests that lower circulating adiponectin concentration is associated with an increased risk of GDM among women with BMI ≥30 kg/m² before or at the point of diagnosis. The GRADE certainty assessment of meta-analyzed evidence was low.

### Inflammatory markers

**Figure 5:**
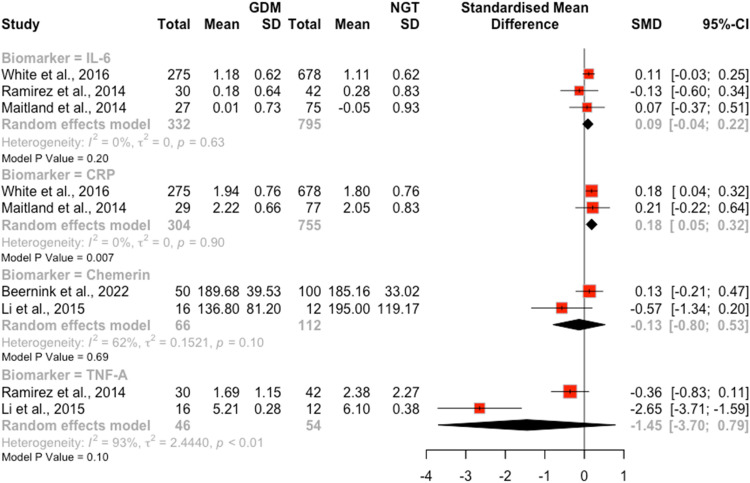
Forest plots of standardised mean differences in log interleukin-6 (Il-6), log c-reactive protein (CRP), chemerin, and TNF-⍺ in pregnant women with a BMI ≥ 30 kg/m^2^ who experience gestational diabetes and those remaining glucose tolerant

7 studies (n=1520) reported on associations between inflammatory markers and the risk of GDM, including 4 secondary analyses of the UPBEAT trial. Biomarkers were assayed prior to or at GDM diagnosis in all studies apart from one.^58^ Elevated CRP was associated with an increased risk of developing GDM v. NGT (*P*=.007; Figure 5). There were no associations between the risk of developing GDM and concentrations of chemerin, TNF-⍺, or Il-6 in GDM v. NGT groups (Figure 5).

Wishlade et al. Biomarkers predicting adverse pregnancy outcomes in women living with obesity. Am J Obstet Gynecol 2025.

*Narrative synthesis of additional studies:* Two studies drawn from the UPBEAT population^49^^,^^51^ demonstrated elevated GlycA was associated with an increased risk of developing GDM, both in early pregnancy and at diagnosis. There was equivocal evidence on the association between IL-6 and the risk of developing GDM.

With the possible exception of CRP and GlycA, there was no evidence of association between the levels of inflammatory markers and risk of GDM in women living with obesity. The GRADE certainty assessment of evidence was very low.

### Lipids

14 studies (n=1821) reported associations between circulating lipid concentrations and the risk of GDM in women living with obesity. Of these, 7 studies utilised data from the UPBEAT trial. Across studies, a range of different lipid species were assayed pre-, post- and at the point of diagnosis.

#### Cholesterol

**Figure 6:**
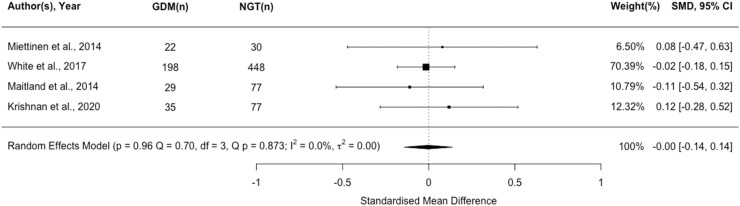
Forest plot of standardised mean difference in log total cholesterol < 24 weeks gestation between pregnant women with a BMI ≥ 30 kg/m^2^ who experience gestational diabetes and those remaining glucose tolerant

Ten studies (n=1684) examined the relationship between cholesterol and risk of GDM. There was no association between total cholesterol measured <24 wks gestation and risk of GDM in women living with obesity (Figure 6), however there was some evidence to suggest that lower total cholesterol measured >24 weeks’ gestation may be associated with increased risk of GDM (Supplementary Figure 3). There was no association between measurements of HDL- or LDL-cholesterol levels and risk of developing GDM in women living with obesity (Supplementary Table 6).

Wishlade et al. Biomarkers predicting adverse pregnancy outcomes in women living with obesity. Am J Obstet Gynecol 2025.

#### Narrative synthesis of additional studies

Three prospective studies drawn from the UPBEAT trial, each using different subsets of the study population, showed conflicting evidence regarding associations between total cholesterol and risk of GDM.^48^^,^^49^^,^^51^ Two smaller single-site prospective cohort studies carried out in predominantly white Irish^59^ and Hispanic^56^ participants respectively, showed significantly lower levels of HDL in women who developed GDM v. NGT before or at diagnosis. This finding is also reflected in a larger cohort study (n=867^29^) drawn from UPBEAT (*P*=.02), although it was not observed in a smaller Finnish study.^60^ There was no association between LDL cholesterol concentrations and GDM risk.^48^^,^^60^

Total or LDL cholesterol concentration is unlikely to be associated with increased risk of GDM in women with BMI ≥30 kg/m². There is equivocal evidence regarding an association between lower HDL cholesterol and increased risk of developing GDM in women with BMI ≥30 kg/m². The certainty of evidence is very low on GRADE analysis.

#### Triglycerides

**Figure 7:**
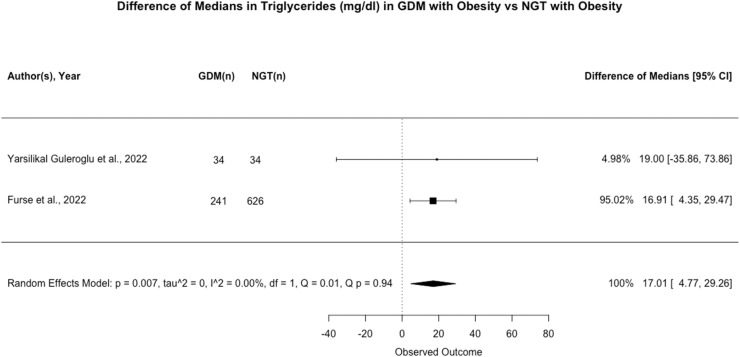
Difference of Medians in Triglycerides (mg/dl) between pregnant women with a BMI ≥ 30 kg/m^2^ who experience gestational diabetes and those remaining glucose tolerant

Of the 14 studies (n=1821) that assayed circulating triglyceride concentrations, only 2 presented data suitable for meta-analysis.^48^^,^^61^ Both studies measured triglyceride concentrations in fasted samples. In these studies, higher triglyceride concentrations were associated with an increased risk of developing GDM (*P*=.007; Figure 7).

Wishlade et al. Biomarkers predicting adverse pregnancy outcomes in women living with obesity. Am J Obstet Gynecol 2025.

#### Narrative synthesis of additional studies

A single-site cohort study (n=72^56^) reported an association between higher triglyceride concentrations and increased risk of developing GDM. Three studies (n=300,^53^ n=646,^49^ n=1303,^41^ respectively) drawn from the UPBEAT trial also found a positive association between circulating triglyceride concentrations and GDM risk. In contrast, 5 further studies – 1 case control and 4 cohort - all reported no association between circulating triglycerides and GDM risk.^57^, ^58^, ^59^, ^60^^,^^62^ Two of these studies assayed triglycerides before or at diagnosis,^59^^,^^62^ two post diagnosis,^57^^,^^58^ and one in each trimester.^60^ However, associations between higher levels of individual lipid species^48^^,^^63^ and lipoprotein composition^49^ and risk of GDM were observed.

There is mixed evidence regarding the association between triglyceride and individual lipid concentrations and risk of GDM in women living with obesity. The certainty of evidence is very low on GRADE analysis.

### Further biomarkers

Other individual studies examined the association between GDM and various other biomarkers, including hemoglobin, hematocrit, mitochondrial DNA, and hepcidin^64^; retinol-binding protein 4^65^; the Quantose IR test^66^; sulphated progesterone metabolites^67^; lipohydroperoxides, malondialdehyde, and protein carbonylation^68^; magnesium^69^; 25 hydroxyvitamin D^70^; plasma glycated PC59 ^38^^,^^71^; bile acids ^52^; PAPP-A, IGF-1, leptin, adipsin and resistin^57^; and the CREBRF rs373863828 allele.^72^ The results of these studies are presented in Supplementary Table 2.

### GDM biomarkers summary

In women living with obesity, our meta-analyses suggests that increased insulin and decreased adiponectin concentrations in the maternal circulation are associated with increased GDM risk. Further evidence is needed to assess the association between risk of GDM and other potential biomarkers (CRP, GlycA, total cholesterol, triglycerides; Supplementary Table 6).

#### Pre-eclampsia

13 studies (n=4593) reported on pre-eclampsia as a pregnancy outcome in women living with obesity. There was considerable heterogeneity between studies in both the definition of pre-eclampsia and the gestation of biomarker measurement (Supplementary Table 2).

### Adipokines

**Figure 8:**
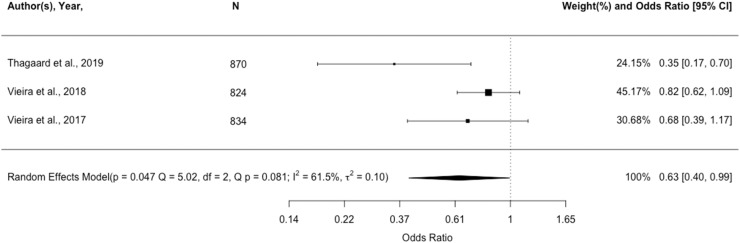
Forest plot showing odds ratios for risk of pre-eclampsia with a unit increase in adiponectin in pregnant women with a BMI ≥30 kg/m²

Four studies (n=2970) assessed the relationship between pre-eclampsia risk and adipokine concentration, three of which were suitable for combining in a meta-analysis. Lower adiponectin was associated with increased risk of pre-eclampsia (*P*=.047, Figure 8).

Wishlade et al. Biomarkers predicting adverse pregnancy outcomes in women living with obesity. Am J Obstet Gynecol 2025.

*Narrative synthesis of additional studies:* A single-centre prospective cohort study from Denmark (n=1292^73^) found lower leptin levels in women with BMI ≥35 kg/m² who developed pre-eclampsia, but no relationship in those with BMI 30 to 35 kg/m². A smaller study^74^ showed an association of between lower visfatin and resistin concentrations and pre-eclampsia, but the biomarkers were assayed only after disease onset.

Evidence suggests that lower adiponectin levels are associated with an increased risk of pre-eclampsia in women living with obesity. However, the certainty of evidence is low on GRADE analysis. Further research is required to explore further potential associations between pre-eclampsia and other adipokines.

### Markers of angiogenesis

Four studies (n=2330) explored associations between angiogenic biomarkers and risk of pre-eclampsia. Due to heterogeneity in data presentation, no meta-analyses could be performed.

Lower concentrations of placental growth factor (PlGF) were associated with increased risk of pre-eclampsia in two prospective cohorts and one case control study (n=824,^75^ n=834,^76^ and n=312,^77^ respectively). All samples were assayed prior to pre-eclampsia onset (<19 weeks’ gestation). A case control study drawn from the multicenter Finnish Genetics of Pre-eclampsia Consortium (FINNPEC) multicenter study^78^ which assayed PlGF in 51 women with obesity also found a negative association between concentration of PlGF and risk of pre-eclampsia, with the result approaching statistical significance with samples taken in the first trimester (*P*=.07), but not with those assayed in the third trimester (*P*=.87). The same study found no association between endoglin and pre-eclampsia risk. No further associations between other angiogenic biomarkers and risk of pre-eclampsia were found in a multinational prospective cohort study excluding women at high risk for pre-eclampsia (n=834) assaying samples <17 weeks’ gestation.^75^

The evidence suggests that higher PlGF concentrations are associated with decreased risk of pre-eclampsia in women living with obesity, at least prior to diagnosis. There is currently insufficient evidence to draw conclusions regarding the relationship of further angiogenic markers to pre-eclampsia.

### Inflammatory markers

4 studies (n=1365) reported on the relationship between inflammatory markers and risk of pre-eclampsia. Due to heterogeneity in data presentation, no meta-analyses could be performed.

No associations between concentration of CRP and risk of pre-eclampsia were observed in either a Finnish case control study (n=360^78^) or a cohort study from the UK (n=824^76^). However the same cohort study reported increased Il-6 associated with increased risk of pre-eclampsia.^76^ A prospective cohort study (n= 116) suggested that complement activation fragments Bb/C3a in the top quartile at <20 weeks’ gestation was associated with increased of developing pre-eclampsia in women living with obesity (*P*<.001).^79^ A single-centre case-control study (n=65) suggested that increased concentrations of IL-6, IFN-*γ*, Interleukin-1b and Il-2 were associated with increased risk of pre-eclampsia, but these were measured after the time of diagnosis.^74^

There is thus no clear evidence that concentrations of CRP or IL-6 are associated with pre-eclampsia prior to diagnosis. The activation of the complement system may be associated with pre-eclampsia prior to disease onset, however further evidence is needed regarding this association.

### Further biomarkers

Other individual studies examined the association between pre-eclampsia and the following biomarkers; homocysteine^80^; 8-isoprostane^81^; kisspeptin^82^; and lipids.^83^, ^84^, ^85^ The results are presented in Supplementary Table 2.

### Pre-eclampsia biomarkers summary

In women living with obesity, our meta-analyses suggest that lower circulating adiponectin concentrations and higher circulating PlGF concentrations are associated with an increased risk of pre-eclampsia. There is insufficient evidence to draw conclusions regarding the association of other potential inflammatory, angiogenic and adipokine biomarkers and pre-eclampsia risk.

#### High birthweight

**Figure 9:**
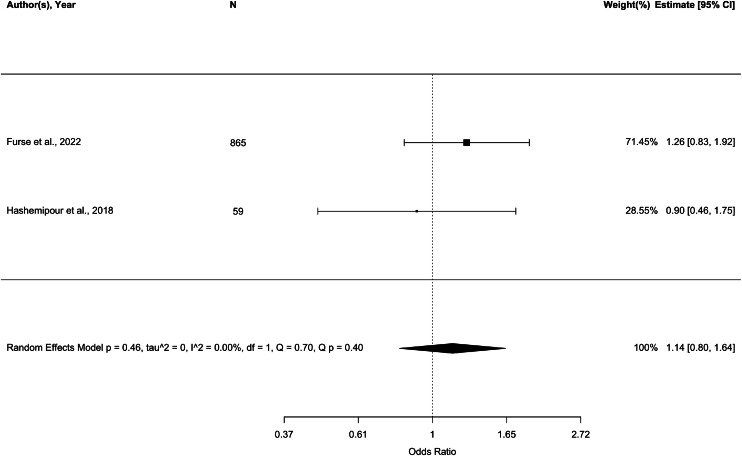
Forest plot showing odds ratio of high birthweight with a unit increase of triglycerides in women with a BMI ≥ 30 kg/m^2^.

4 studies (n=1416) reported on metrics of high birthweight in women living with obesity. 2 studies reported the outcome of large-for-gestational age (LGA).^48^^,^^85^ 2 studies reported macrosomia, defined as birthweight ≥4000 g in one study^86^ and >4500 g or >95^th^ centile in the other.^87^ 3 cohort studies found no association between triglyceride levels and risk of high birthweight,^48^^,^^85^^,^^86^ 2 of which were included in a meta-analysis (Figure 9). There was no association between lipogenesis-related di- and tri-glycerides and risk of LGA risk in a secondary analysis of UPBEAT data.^48^ One further prospective cohort study (n=105) found no association between insulin or glucose concentrations and risk of LGA.^87^ No other biomarkers were reported with respect to high birthweight. There was no evidence of an association between any assayed biomarker and high birthweight in women living with obesity.

Wishlade et al. Biomarkers predicting adverse pregnancy outcomes in women living with obesity. Am J Obstet Gynecol 2025.

#### Other outcomes

Data were available for 4 of our other prespecified outcomes: preterm birth (PTB), small for gestational age (SGA), gestational hypertension (GH), and hypertensive disorders of pregnancy (HDP). None of these outcomes had sufficient data in an appropriate form for meta-analysis.

### Preterm birth

Four studies (n=325) reported rates of PTB. Definitions of PTB varied by gestational week of delivery and whether the delivery was spontaneous (sPTB) or iatrogenic (iPTB) (Supplementary Table 2).

A retrospective cohort study containing 34 primiparous participants with obesity showed that women with sPTB <31 wks gestation had a metabolomic profile containing higher concentrations of auto-oxidation metabolites and evidence of dyslipidemia compared to those delivering at term.^88^ A case control study suggested that Interleukin-2 (Il-2) and TNF-⍺ were decreased in women with sPTB <34 wks gestation compared to those who delivered full-term.^89^ A small case control study (n=67) showed Interleukin 2 Receptor Alpha (Il-2RA) and Tumour Necrosis Factor Receptor 1 (TNFR1) were higher, whereas Vascular Endothelial Growth Factor Receptor 3 (VEGFR3) was lower in women with BMI >30 kg/m^2^ who had sPTB <31 wks compared to those who delivered at term.^90^ A nested case control study^91^ (n=53) found Urinary N-acetyl glycoprotein was increased in women with iPTB, although the relationship was not tested for significance.

### Small-for-gestational age (SGA)

One prospective cohort study from the UK and Netherlands, drawn from the Vitamins in Pre-eclampsia trial^85^ (n=385) showed that lower vitamin C concentration was associated with an increased risk of SGA v. appropriate for gestational age births. The study found no relationship between maternal triglyceride concentrations and SGA.

### Hypertensive disorders of pregnancy (excluding pre-eclampsia)

Three studies (n=395) reported hypertensive disorders of pregnancy. One case control study (n=101) reported an outcome of gestational hypertension,^42^ finding that women with gestational hypertension had elevated levels of Growth/Differentiation Factor 15 (GDF-15) in the second and third trimesters compared to controls (reported with significance *P*<.05). Other studies found no relationship between either cell-free DNA^92^ or HbA1c^42^ and risk of hypertensive disorders of pregnancy in women living with obesity.

### Other outcome biomarkers summary

Overall, the paucity of evidence means that clear conclusions cannot be drawn regarding associations of biomarkers and adverse pregnancy outcomes other than GDM and pre-eclampsia.

### Comment

#### Principal findings

We identified >500 distinct biomarkers tested for correlation with adverse pregnancy outcomes in women living with obesity. However, heterogeneity in both outcomes and assays meant that meta-analysis was only possible for 13 biomarkers with respect to a limited outcome set of GDM, pre-eclampsia, and high birthweight. Our planned composite adverse outcome could only be evaluated for a single biomarker, adiponectin.

A striking finding was the lack of data available to address our key aim of stratifying risk of adverse pregnancy outcomes in women living with obesity. The largest study in our review included n=1303 participants. The largest number of participants we could include in a single meta-analysis was n=2528, leading to low or very low certainty of evidence associated with all analyses. Women living with obesity represent 16%^93^ of the global pregnancy population, and the greater surveillance, testing, and intervention recommended during their pregnancies represents considerable resource implications for overstretched healthcare systems. A key finding is that more data is required to assess the potential for risk-stratification within a heterogenous pregnant population living with obesity.

#### Comparison with existing literature

Lower levels of adiponectin were associated with poor pregnancy outcomes in women living with obesity, in particular increased risk of pre-eclampsia. In the general pregnancy population, low levels of early-pregnancy adiponectin have been linked to increased risk of both GDM^94^ and preeclampsia.^95^^,^^96^ Further, increased adiponectin has been associated with increased likelihood of positive outcomes in pregnancies with BMI ≥30 kg/m² in a risk prediction model including clinical and biochemical factors.^97^ The moderate effect size of the association between adiponectin and pre-eclampsia specifically in unfasted early-pregnancy samples in women with BMI ≥30 kg/m² suggests a possible route for further research towards developing predictive tests.

Increased insulin was associated with higher risk of developing GDM from up to 10 weeks prior to diagnostic testing in women living with obesity, however the certainty of evidence was very low. This finding aligns with the general pregnancy population, in which higher insulin concentrations <16 weeks’ gestation are associated with later development of GDM.^98^, ^99^, ^100^ There was also an association between reduced PlGF and a higher risk of developing pre-eclampsia, a finding that aligns with meta-analyses in the total pregnancy population.^101^, ^102^, ^103^

#### Potential mechanisms

Pregnancy is a metabolic "stress test" which may exaggerate underlying metabolic dysfunction in women with obesity,^104^ with metabolically unhealthy pregnant women with BMI ≥30 kg/m² at significantly greater risk of poor pregnancy outcomes than those defined as metabolically healthy.^105^ Adipose tissue function correlates with obese metabolic phenotypes.^106^, ^107^, ^108^ Dysfunctional adipose tissue is associated with both decreased circulating adiponectin concentrations^109^ and increased insulin resistance.^110^ Thus, the associations we found between poor pregnancy outcomes and these biomarkers may be indicative of the dysfunctional adipose tissue seen in metabolically unhealthy obese phenotypes.

### Strengths and limitations

Key strengths of the study include our comprehensive search strategy compiled with the aid of an information specialist, the range of biomarkers assayed, and the focus on biomarkers assayable pragmatically during pregnancy. Our search criteria included studies that assayed biomarkers from any kind of biological sample, although all included studies reported biomarkers in maternal serum or plasma.

A major limitation is small number of studies that have addressed adverse outcomes specifically in women living with obesity, and small participant sizes within available studies. Variability in biomarkers measured limited our ability to combine data in meta-analyses. The use of standardized mean difference (SMD) or odds ratio of log data to express results is necessary and robust but limits intuitive interpretation. There were also insufficient data to be able to statistically explore sources of heterogeneity through subgroup analyses. Additional sources of clinical variability included the gestation at which biomarkers were assayed and the criteria used to diagnose outcomes, and when and how BMI was calculated.

## Conclusions and implications

Inflammation,^111^ dyslipidaemia^112^ and insulin resistance^113^ are important metabolic components of obesity. Our study suggests that pregnant women with obesity who experience adverse outcomes have greater metabolic dysfunction than those with uncomplicated pregnancies. It is conceivable that biomarkers of metabolic dysfunction, for example adiponectin, could be used to stratify risk of adverse pregnancy outcomes within obese women but this requires further research. Indeed, the lack of data available in this area is an important and unexpected finding of our review, highlighting a major challenge for the field. In view of the high burden of providing safe maternity care to increasing numbers of women living with obesity globally,^114^ there is an urgent need for further studies investigating reliable identification of those at highest risk of adverse pregnancy outcomes, enabling more effective targeting of maternity care interventions.

## CRediT authorship contribution statement

**Tabitha Wishlade:** Writing – original draft, Formal analysis, Data curation. **Sara Wetzler:** Writing – review & editing, Data curation. **Catherine E. Aiken:** Writing – review & editing, Conceptualization.

## Appendix Supplementary materials

Image, application 1

Image, application 2

Image, application 3

Image, application 4

Image, application 5

Image, application 6

Image, application 7

Image, application 8

Image, application 9

Image, application 10

Image, application 11

## Footnotes

Supplementary material associated with this article can be found in the online version at doi:10.1016/j.xagr.2025.100527.
