# Supplementary material for: Biomarkers predicting adverse pregnancy outcomes in women living with obesity: a systematic review and meta-analysis
Source: AJOG Glob Rep. 2025 Jul 22;5(3):100527. doi: 10.1016/j.xagr.2025.100527 (PMC12465041; doi:10.1016/j.xagr.2025.100527)
Supplement: Supplementary file 1 [file mmc1.docx]

**Appendix A: Search Strategy**

The databases Ovid MEDLINE (1946 to 19/08/2024), Ovid Embase (1974 – 19/08/2024), PubMed (searched 19/08/2024), Scopus (searched 19/08/2024) and CENTRAL (searched 19/08/2024) were searched. No language filters were applied during these searches.

**Pubmed:**

Top of Form

Pubmed

| **Search** | **Actions** | **Details** | **Query** | **Results** |
| --- | --- | --- | --- | --- |
| #5 |  |  | Search: **#1 AND #2 AND #3 AND #4** Sort by: **Most Recent** | [3,145](https://pubmed.ncbi.nlm.nih.gov/?term=%231+AND+%232+AND+%233+AND+%234&sort=date) |
| #4 |  |  | Search: **"Obesity" [Majr] OR "obesity, maternal" [Majr] OR "Body Mass Index" [Majr] OR "maternal obesity" [Title/Abstract] OR "body mass index" [Title/Abstract] OR "obesity" [Title/Abstract] OR "BMI" [Title/Abstract]** Sort by: **Most Recent** | [632,967](https://pubmed.ncbi.nlm.nih.gov/?term=%22Obesity%22+%5BMajr%5D+OR+%22obesity%2C+maternal%22+%5BMajr%5D+OR+%22Body+Mass+Index%22+%5BMajr%5D+OR+%22maternal+obesity%22+%5BTitle%2FAbstract%5D+OR+%22body+mass+index%22+%5BTitle%2FAbstract%5D+OR+%22obesity%22+%5BTitle%2FAbstract%5D+OR+%22BMI%22+%5BTitle%2FAbstract%5D&sort=date) |
| #3 |  |  | Search: **("Pregnancy Outcome" [Majr] OR "diabetes, gestational" [Majr] OR "pre eclampsia" [Majr] OR "hypertension, pregnancy induced"[Majr] OR "Fetal Growth Retardation" [Majr] OR "Polyhydramnios" [Majr] OR "Oligohydramnios" [Majr] OR "fetal membranes, premature rupture" [Majr] OR "Cesarean Section" [Majr] OR "Premature Birth" [Majr] OR "infant, low birth weight" [Majr] OR "infant, small for gestational age" [Majr] OR "infant, large for gestational age" [Majr] OR "Stillbirth" [Majr] OR "Fetal Death" [Majr] OR "Perinatal Death" [Majr] OR "Obstetric Labor, Premature"[Mesh] OR "Labor, Induced"[Mesh] OR "pregnancy-induced hypertension" [Title/Abstract] OR "PIH" [Title/Abstract]OR "fetal growth restriction" [Title/Abstract] OR "FGR" [Title/Abstract] OR "IUGR" [Title/Abstract] OR "Polyhydramnios" [Title/Abstract] OR "Oligohydramnios" [Title/Abstract] OR "premature labour" [Title/Abstract] OR "preterm labour" [Title/Abstract] OR "premature labor" [Title/Abstract] OR "preterm labor" [Title/Abstract] OR "premature rupture of the membranes" [Title/Abstract] OR "preterm rupture of the membranes" [Title/Abstract] OR "induced labour" [Title/Abstract] OR "induced labor" [Title/Abstract] OR "induction of labour" [Title/Abstract] OR "induction of labor" [Title/Abstract] OR "cesarean section" [Title/Abstract] OR "caesarean section" [Title/Abstract] OR "premature birth" [Title/Abstract] OR "preterm birth" [Title/Abstract] OR "preterm delivery" [Title/Abstract]OR "premature delivery" [Title/Abstract] OR "low birthweight" [Title/Abstract] OR "LBW" [Title/Abstract] OR "small for gestational age" [Title/Abstract] OR "SGA" [Title/Abstract] OR "Pregnancy Outcome" [Title/Abstract] OR "gestational diabetes" [Title/Abstract] OR "GDM" [Title/Abstract] OR "preeclampsia" [Title/Abstract] OR "PET" [Title/Abstract] OR "gestational hypertension" [Title/Abstract] OR "large for gestational age" [Title/Abstract] OR "LGA" [Title/Abstract] OR "macrosomia" [Title/Abstract] OR "Stillbirth" [Title/Abstract] OR "Fetal Death" [Title/Abstract] OR "FDIU" [Title/Abstract] OR "IUD" [Title/Abstract] OR "fetal demise" [Title/Abstract] OR "perinatal death" [Title/Abstract])** Sort by: **Most Recent** | [446,575](https://pubmed.ncbi.nlm.nih.gov/?term=longquery360046a1180b2b5bdb71&sort=date) |
| #2 |  |  | Search: **"Biomarkers" [Majr] OR "Lipids" [Majr] OR "Proteins"[Majr] OR biomarker*[Title/Abstract] OR lipid* [Title/Abstract] OR protein*[Title/Abstract]** Sort by: **Most Recent** | [7,797,591](https://pubmed.ncbi.nlm.nih.gov/?term=%22Biomarkers%22+%5BMajr%5D+OR+%22Lipids%22+%5BMajr%5D+OR+%22Proteins%22%5BMajr%5D+OR+biomarker%2A%5BTitle%2FAbstract%5D+OR+lipid%2A+%5BTitle%2FAbstract%5D+OR+protein%2A%5BTitle%2FAbstract%5D&sort=date) |
| #1 |  |  | Search: **(pregnan*[Title/Abstract] OR 'Pregnancy' [Majr])** Sort by: **Most Recent** | [710,967](https://pubmed.ncbi.nlm.nih.gov/?term=%28pregnan%2A%5BTitle%2FAbstract%5D+OR+%27Pregnancy%27+%5BMajr%5D%29&sort=date) |

Showing 1 to 5 of 5 entries

**Ovid Embase:**

1 exp *obesity/ 310078

2 obes*.ti,ab. 599185

3 exp *maternal obesity/ 3459

4 maternal obesity.ti,ab. 5672

5 exp *body mass/ 44142

6 body mass index.ti,ab. 370441

7 BMI.ti,ab. 439883

8 exp *biological marker/ 139881

9 biomarker*.ti,ab. 661834

10 exp *lipid/ 775926

11 lipid*.ti,ab. 774538

12 exp *protein/ 152518

13 protein*.ti,ab. 4417361

14 marker*.ti,ab. 1409208

15 metabolic.ti,ab. 905533

16 biomolecular.ti,ab. 20357

17 exp *pregnancy outcome/ 29864

18 pregnancy outcome.ti,ab. 21111

19 exp *pregnancy diabetes mellitus/ 4459

20 gestational diabetes.ti,ab. 36268

21 GDM.ti,ab. 19608

22 exp *preeclampsia/ 34100

23 preeclampsia.ti,ab. 47465

24 pre-eclampsia.ti,ab. 19228

25 PET.ti,ab. 239783

26 exp *maternal hypertension/ 45166

27 pregnancy-induced hypertension.ti,ab. 6286

28 gestational hypertension.ti,ab. 7210

29 PIH.ti,ab. 3926

30 exp *intrauterine growth retardation/ 14231

31 fetal growth restriction.ti,ab. 10258

32 FGR.ti,ab. 4927

33 IUGR.ti,ab. 11200

34 exp *hydramnios/ 60

35 polyhydramnios.ti,ab. 4408

36 exp *oligohydramnios/ 1205

37 oligohydramnios.ti,ab. 4547

38 exp *premature labor/ 79482

39 premature labour.ti,ab. 1051

40 preterm labour.ti,ab. 3658

41 premature labor.ti,ab. 2415

42 preterm labor.ti,ab. 10400

43 exp *premature fetus membrane rupture/ 3312

44 preterm rupture of membranes.ti,ab. 561

45 premature rupture of membranes.ti,ab. 6988

46 PROM.ti,ab. 8540

47 exp *labor induction/ 7515

48 induction of labour.ti,ab. 4179

49 induction of labor.ti,ab. 5679

50 induced labour.ti,ab. 527

51 induced labor.ti,ab. 1049

52 exp *cesarean section/ 36790

53 cesarean section.ti,ab. 41302

54 caesarean section.ti,ab. 33440

55 exp *prematurity/ 61272

56 premature birth.ti,ab. 6297

57 preterm birth.ti,ab. 36911

58 preterm delivery.ti,ab. 19219

59 premature delivery.ti,ab. 4449

60 exp *low birth weight/ 22545

61 low birthweight.ti,ab. 10645

62 LBW.ti,ab. 7506

63 exp *small for gestational age/ 493

64 small for gestational age.ti,ab. 19147

65 SGA.ti,ab. 18151

66 exp *macrosomia/ 1869

67 macrosomia.ti,ab. 7377

68 exp *large for gestational age/ 845

69 large for gestational age.ti,ab. 5261

70 LGA.ti,ab. 5476

71 exp *stillbirth/ 5255

72 stillbirth.ti,ab. 16273

73 exp *fetus death/ 11992

74 fetal death in utero.ti,ab. 295

75 FDIU.ti,ab. 36

76 IUD.ti,ab. 8896

77 exp *perinatal death/ 2241

78 perinatal death.ti,ab. 4705

79 exp *newborn death/ 1412

80 newborn death.ti,ab. 214

81 1 or 2 or 3 or 4 or 5 or 6 or 7 1077808

82 8 or 9 or 10 or 11 or 12 or 13 or 14 or 15 or 16 7409340

83 17 or 18 or 19 or 20 or 21 or 22 or 23 or 24 or 25 or 26 or 27 or 28 or 29 or 30 or 31 or 32 or 33 or 34 or 35 or 36 or 37 or 38 or 39 or 40 or 41 or 42 or 43 or 44 or 45 or 46 or 47 or 48 or 49 or 50 or 51 or 52 or 53 or 54 or 55 or 56 or 57 or 58 or 59 or 60 or 61 or 62 or 63 or 64 or 65 or 66 or 67 or 68 or 69 or 70 or 71 or 72 or 73 or 74 or 75 or 76 or 77 or 78 or 79 or 80 664959

84 81 and 82 and 83 11901

85 exp *pregnancy/ 215928

86 pregnan*.ti,ab. 809417

87 85 or 86 840907

88 84 and 87 7265

89 8 or 9 or 10 or 11 or 12 or 13 or 14 6833554

90 81 and 83 and 87 and 89 5142

**CENTRAL:**

#1 (( "biomarker*" OR "lipid*" OR "protein*" ) AND ( "pregnancy" OR "pregnant" ) AND ( "maternal obesity" OR "Body Mass Index" OR "Obesity" OR "BMI" ) AND ( "pregnancy-induced hypertension" OR "PIH" OR "fetal growth restriction" OR "FGR" OR "IUGR" OR "Polyhydramnios" OR "Oligohydramnios" OR "premature labour" OR "preterm labour" OR "premature labor" OR "preterm labor" OR "premature rupture of the membranes" OR "preterm rupture of the membranes" OR "induced labour" OR "induced labor" OR "induction of labour" OR "induction of labor" OR "Cesarean section" OR "Caesarean section" OR "premature birth" OR "preterm birth" OR "preterm delivery" OR "premature delivery" OR "low birthweight" OR "LBW" OR "small for gestational age" OR "SGA" OR " pregnancy outcome " OR " gestational diabetes " OR " gdm " OR " pre eclampsia " OR " preeclampsia " OR "PET" OR " gestational hypertension " OR " large for gestational age " OR " macrosomia " OR "LGA" OR " stillbirth " OR " fetal death " OR " FDIU " OR " IUD " OR "fetal demise" OR "perinatal death" )):ti,ab,kw (Word variations have been searched) 475

**SCOPUS:**

TITLE-ABS ( ( ( "biomarker*" OR "lipid*" OR "protein*" ) AND ( "pregnancy" OR "pregnant" ) AND ( "maternal obesity" OR "Body Mass Index" OR "Obesity" OR "BMI" ) AND ( "pregnancy-induced hypertension" OR "PIH" OR "fetal growth restriction" OR "FGR" OR "IUGR" OR "Polyhydramnios" OR "Oligohydramnios" OR "premature labour" OR "preterm labour" OR "premature labor" OR "preterm labor" OR "premature rupture of the membranes" OR "preterm rupture of the membranes" OR "induced labour" OR "induced labor" OR "induction of labour" OR "induction of labor" OR "Cesarean section" OR "Caesarean section" OR "premature birth" OR "preterm birth" OR "preterm delivery" OR "premature delivery" OR "low birthweight" OR "LBW" OR "small for gestational age" OR "SGA" OR " pregnancy outcome " OR " gestational diabetes " OR " gdm " OR " pre eclampsia " OR " preeclampsia " OR "PET" OR " gestational hypertension " OR " large for gestational age " OR " macrosomia " OR "LGA" OR " stillbirth " OR " fetal death " OR " FDIU " OR " IUD " OR "fetal demise" OR "perinatal death" ) ) ) 2288

**Ovid MEDLINE:**

1 exp *Obesity/ 184656

2 obesity.ti,ab. 326132

3 obese.ti,ab. 157670

4 exp *Obesity, Maternal/ 644

5 maternal obesity.ti,ab. 3439

6 exp *Body Mass Index/ 24608

7 body mass index.ti,ab. 252697

8 BMI.ti,ab. 208778

9 exp *Biomarkers/ 296281

10 biomarker*.ti,ab. 445546

11 exp *Lipids/ 757885

12 lipid*.ti,ab. 625348

13 exp *Proteins/ 5110685

14 protein*.ti,ab. 3680404

15 marker*.ti,ab. 976033

16 exp *Pregnancy Outcome/ 38818

17 pregnancy outcome*.ti,ab. 33884

18 exp *Diabetes, Gestational/ 16052

19 gestational diabetes.ti,ab. 23079

20 GDM.ti,ab. 12605

21 exp *Pre-Eclampsia/ 29546

22 preeclampsia.ti,ab. 29284

23 pre-eclampsia.ti,ab. 12975

24 PET.ti,ab. 135985

25 exp *Hypertension, Pregnancy-Induced/ 36017

26 pregnancy-induced hypertension.ti,ab. 4393

27 gestational hypertension.ti,ab. 4388

28 PIH.ti,ab. 2520

29 exp *Fetal Growth Retardation/ 11910

30 fetal growth restriction.ti,ab. 6421

31 FGR.ti,ab. 3004

32 exp *Polyhydramnios/ 1100

33 polyhydramnios.ti,ab. 2908

34 exp *Oligohydramnios/ 818

35 oligohydramnios.ti,ab. 3018

36 Obstetric Labor, Premature/ 13924

37 premature labour.ti,ab. 816

38 premature labor.ti,ab. 2358

39 preterm labour.ti,ab. 2125

40 preterm labor.ti,ab. 7146

41 exp *Fetal Membranes, Premature Rupture/ 5904

42 premature rupture of the membranes.ti,ab. 6123

43 preterm rupture of the membranes.ti,ab. 446

44 PROM.ti,ab. 5263

45 exp *Labor, Induced/ 6954

46 induced labour.ti,ab. 365

47 induced labor.ti,ab. 871

48 induction of labour.ti,ab. 2516

49 induction of labor.ti,ab. 4043

50 exp *Cesarean Section/ 28483

51 cesarean section.ti,ab. 31444

52 caesarean section.ti,ab. 20021

53 exp *Premature Birth/ 17376

54 premature birth.ti,ab. 4268

55 preterm birth.ti,ab. 25219

56 preterm delivery.ti,ab. 12424

57 premature delivery.ti,ab. 3086

58 exp *Infant, Low Birth Weight/ 20518

59 low birthweight.ti,ab. 8823

60 LBW.ti,ab. 5414

61 exp *Infant, Small for Gestational Age/ 4081

62 small for gestational age.ti,ab. 13909

63 SGA.ti,ab. 11338

64 exp *Fetal Macrosomia/ 1846

65 macrosomia.ti,ab. 4787

66 large for gestational age.ti,ab. 3681

67 LGA.ti,ab. 3506

68 exp *Stillbirth/ 3866

69 stillbirth.ti,ab. 11378

70 exp *Fetal Death/ 13785

71 fetal death in utero.ti,ab. 239

72 FDIU.ti,ab. 10

73 IUD.ti,ab. 7876

74 exp *Perinatal Death/ 1742

75 perinatal death.ti,ab. 3480

76 newborn death.ti,ab. 153

77 1 or 2 or 3 or 4 or 5 or 6 or 7 or 8 652659

78 16 or 17 or 18 or 19 or 20 or 21 or 22 or 23 or 24 or 25 or 26 or 27 or 28 or 29 or 30 or 31 or 32 or 33 or 34 or 35 or 36 or 37 or 38 or 39 or 40 or 41 or 42 or 43 or 44 or 45 or 46 or 47 or 48 or 49 or 50 or 51 or 52 or 53 or 54 or 55 or 56 or 57 or 58 or 59 or 60 or 61 or 62 or 63 or 64 or 65 or 66 or 67 or 68 or 69 or 70 or 71 or 72 or 73 or 74 or 75 or 76 443493

79 9 or 10 or 11 or 12 or 13 or 14 or 15 8238400

80 exp *Pregnancy/ 166670

81 pregnan*.ti,ab. 622354

82 80 or 81 693536

83 77 and 78 and 79 and 82 3574

84 limit 83 to dt=19460101-20240819 3569
