# Supplementary material for: Biomarkers predicting adverse pregnancy outcomes in women living with obesity: a systematic review and meta-analysis
Source: AJOG Glob Rep. 2025 Jul 22;5(3):100527. doi: 10.1016/j.xagr.2025.100527 (PMC12465041; doi:10.1016/j.xagr.2025.100527)
Supplement: Supplementary file 2 [file mmc2.docx]

**Appendix 2: Variables for which data was extracted from publications included in the review**

General:

First author

Publication year

Paper title

Country

Language

Study Methods:

Study aim

Study design

Number of sites

Setting

Recruitment process

Enrolment start/end dates

Statistical methods

Methods used to account for missing values

If/how study was powered

If/how gestational age of sample was accounted for

Blinded or unblinded

Participants

Number affected by outcome

Number unaffected by outcome

Withdrawals/loss to follow up

Inclusion and exclusion criteria

Definition of obesity used

When/how BMI was measured

Average BMI and age in affected and unaffected groups

Ethnicity, parity, smoking, pregnancy complications and underlying comorbidities in affected and unaffected groups

Intervention

Biomarkers assayed

Method of sampling

If samples were fasted

Method of biomarker measurement

Gestation of assay

Limits of detection

Biomarker quantity in affected and unaffected groups

If/how biomarker data was transformed

Units of biomarker measurement

Outcomes

Gestation of outcome diagnosis

Diagnostic criteria used

Adjusted and unadjusted odds ratios or raw data for 2x2 table
