# Supplementary material for: Biomarkers predicting adverse pregnancy outcomes in women living with obesity: a systematic review and meta-analysis
Source: AJOG Glob Rep. 2025 Jul 22;5(3):100527. doi: 10.1016/j.xagr.2025.100527 (PMC12465041; doi:10.1016/j.xagr.2025.100527)
Supplement: Supplementary file 3 [file mmc3.docx]

**Supplementary Figure 1: Assessment of Small Study Effects (Funnel Plot and Trim-and-Fill) of Meta-Analyses containing ≥3 Studies**

1. Adiponectin and Composite Poor Pregnancy Outcome:


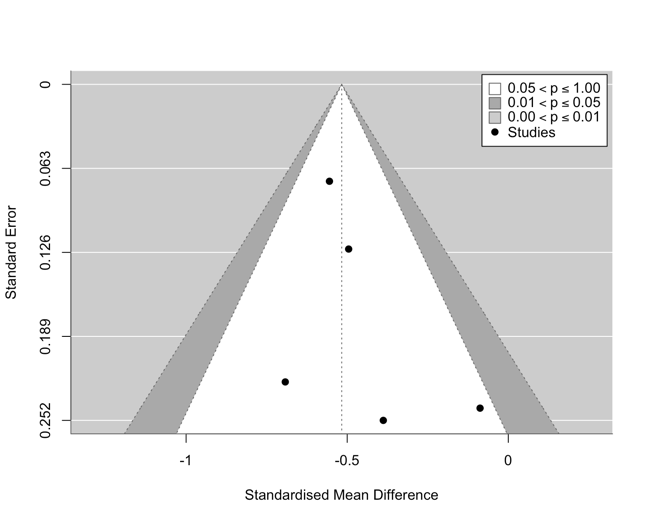


Supplementary Figure 1.1: Funnel plot showing small study effects in adiponectin-poor pregnancy outcome association.


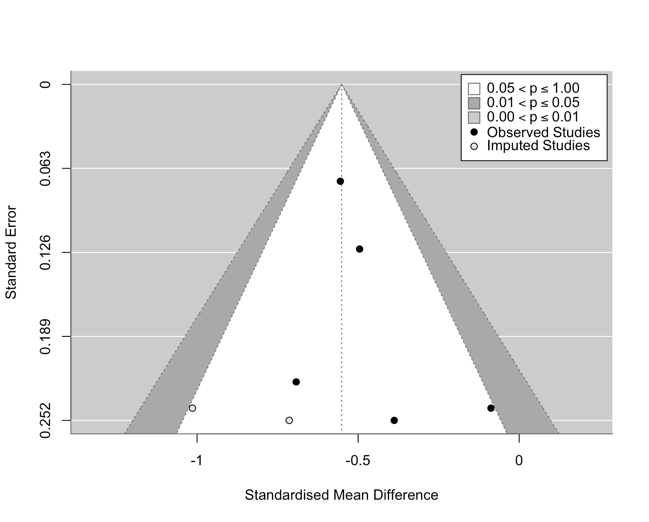


Supplementary Figure 1.2: Trim-and-Fill plot showing the summary effect of the adiponectin-poor pregnancy outcome association adjusted for publication bias.

1. Leptin and Composite Poor Outcome


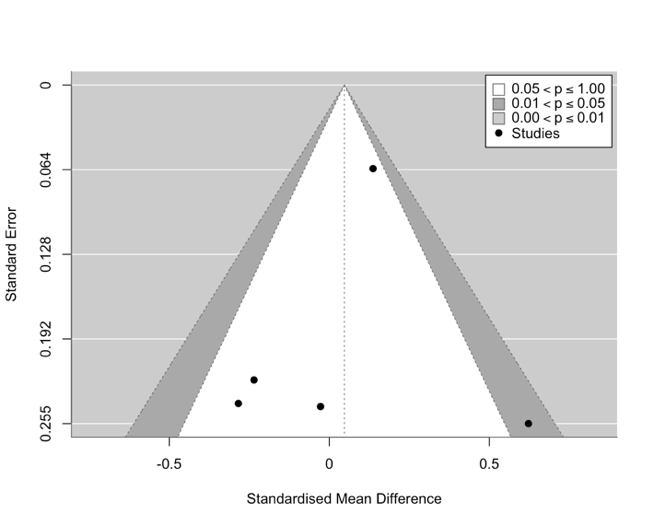


Supplementary Figure 1.3: Funnel plot showing small study effects in leptin-poor outcome association.


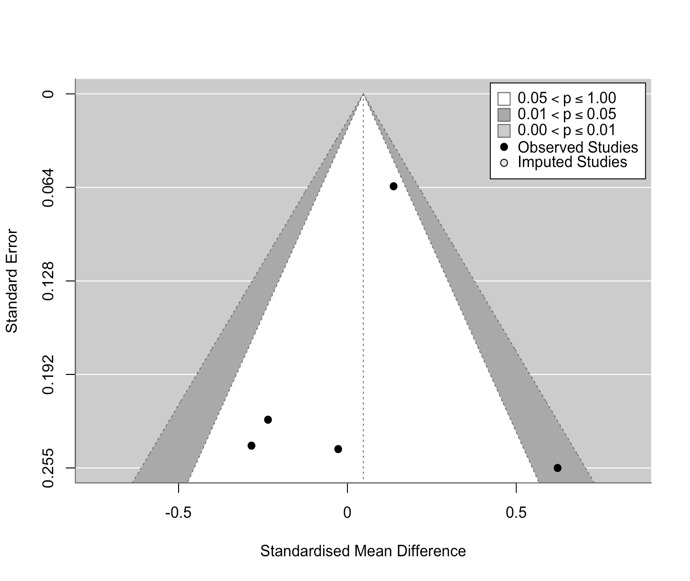


Supplementary Figure 1.4: Trim-and-Fill plot showing the summary effect of the leptin-poor outcome association adjusted for publication bias.

1. Insulin and Gestational Diabetes Mellitus (GDM)


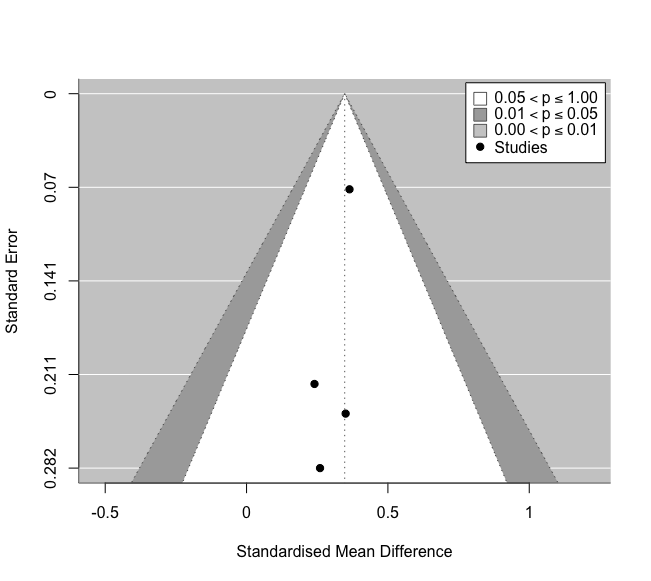


Supplementary Figure 1.5: Funnel plot showing small study effects in insulin-GDM association.


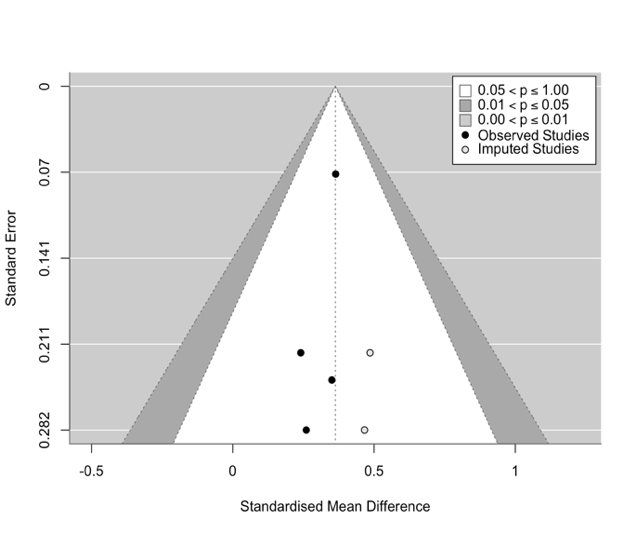


Supplementary Figure 1.6: Trim-and-Fill plot showing the summary effect of the insulin-GDM association adjusted for publication bias.

1. Interleukin-6 (Il-6) and GDM


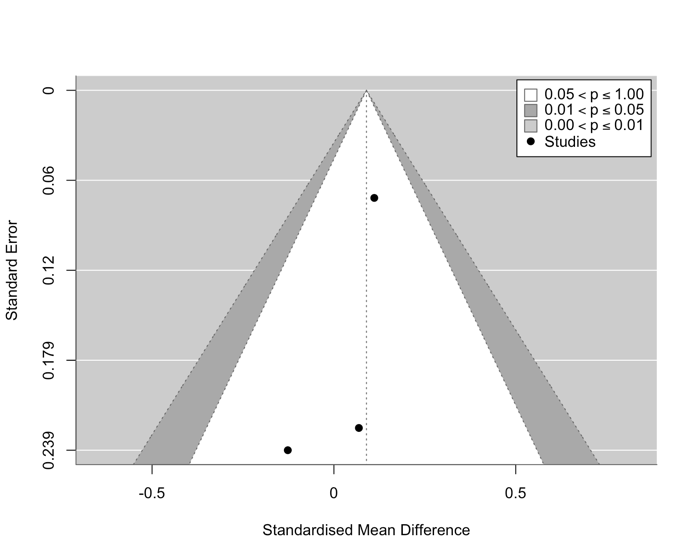


Supplementary Figure 1.7: Funnel plot showing small study effects in IL6-GDM association.


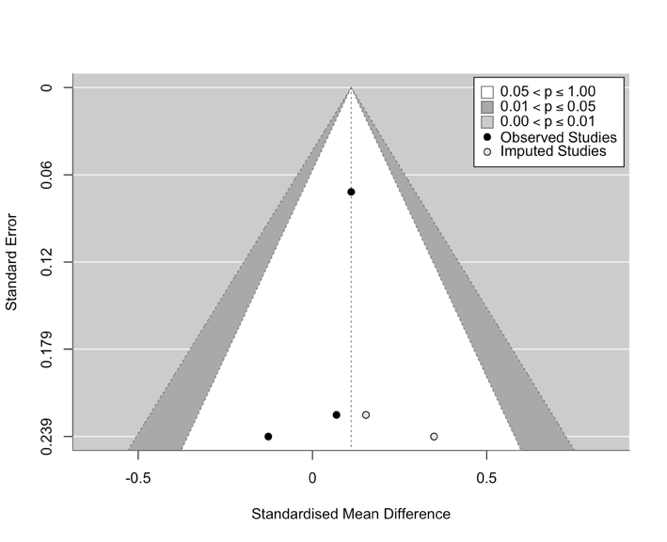


Supplementary Figure 1.8: Trim-and-Fill plot showing the summary effect of the Il-6-GDM association adjusted for publication bias.

1. Total Cholesterol <24/40 and GDM


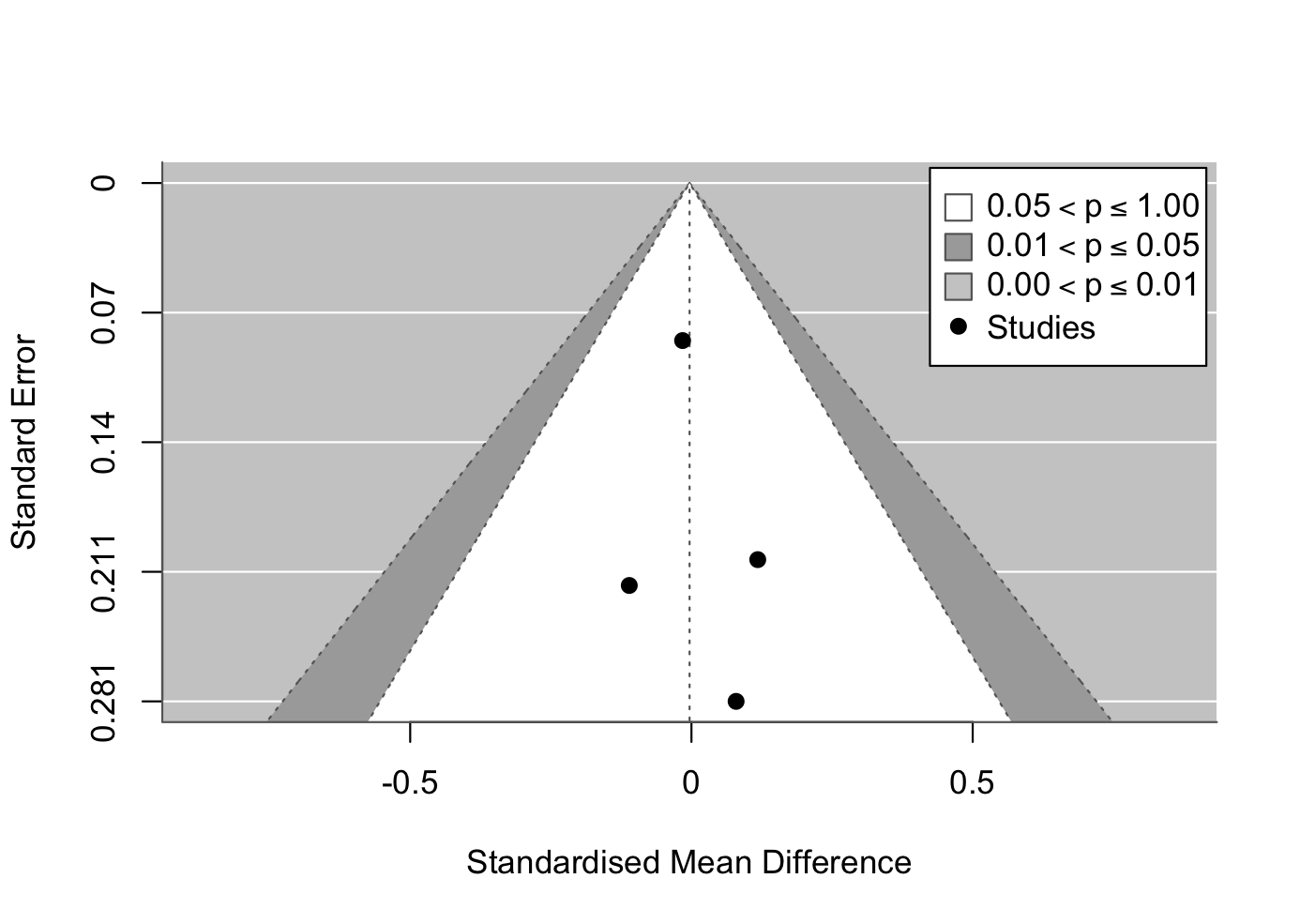


Supplementary Figure 1.9: Funnel plot showing small study effects in total cholesterol < 24/40-GDM association.


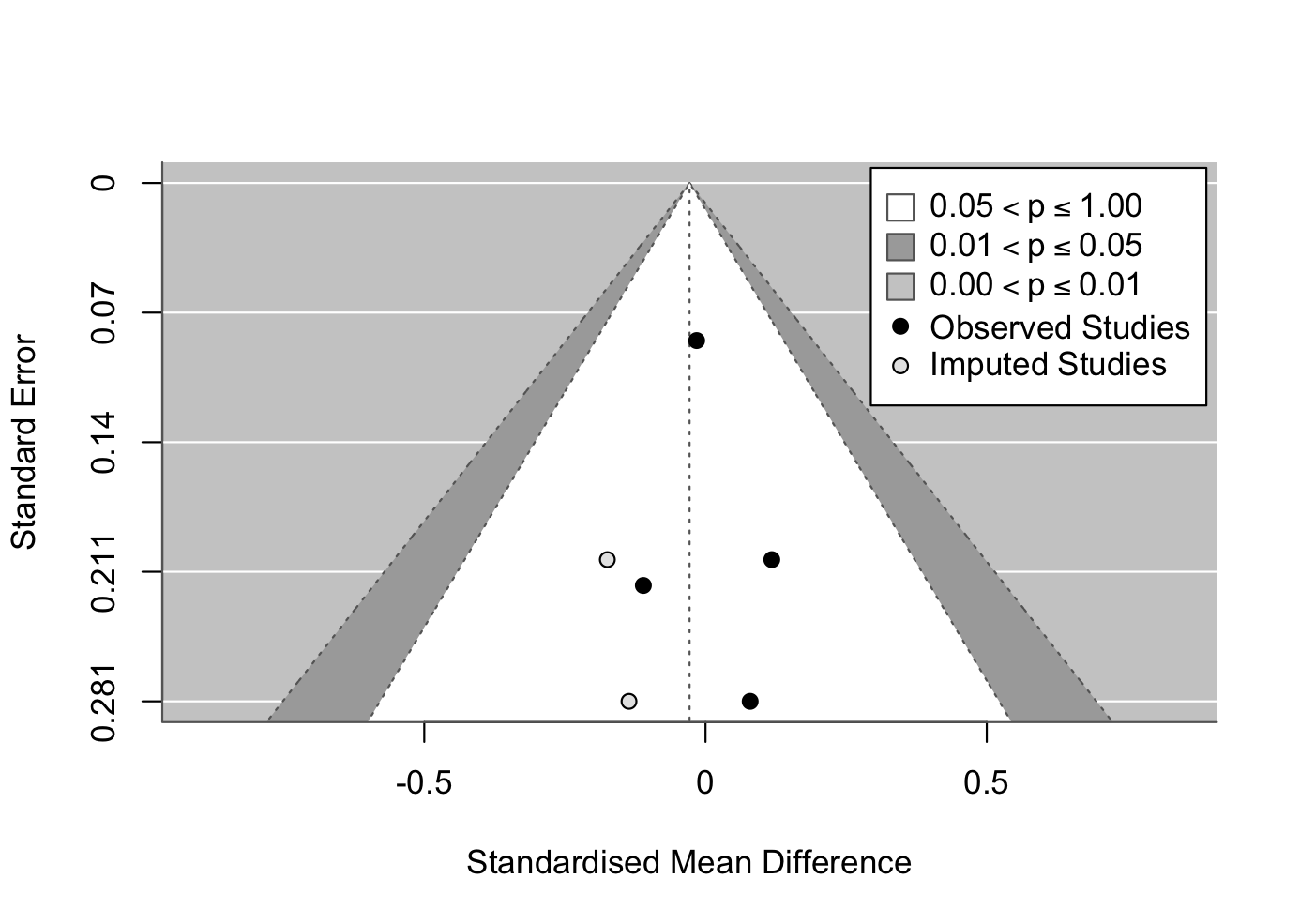


Supplementary Figure 1.10: Trim-and-Fill plot showing the summary effect of the total cholesterol <24/40-GDM association adjusted for publication bias.

1. Total Cholesterol >24/40 and GDM


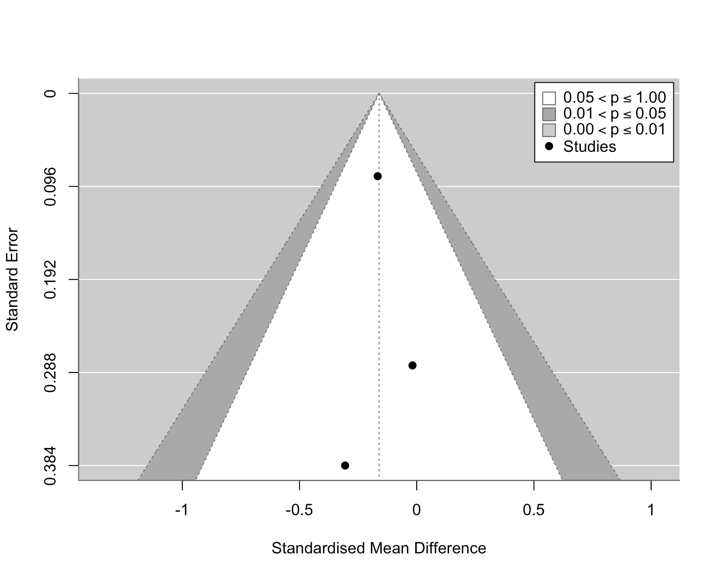


Supplementary Figure 1.11: Funnel plot showing small study effects in total cholesterol ≥24/40-GDM association.


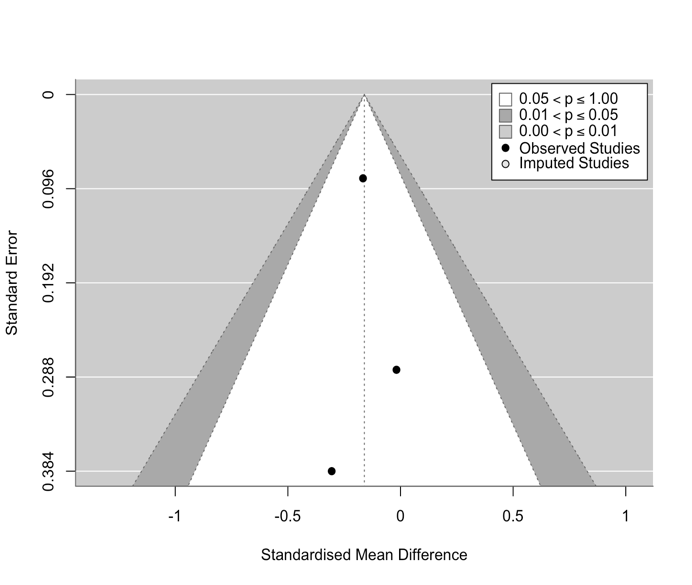


Supplementary Figure 1.12: Trim-and-Fill plot showing the summary effect of the total cholesterol ≥24/40-GDM association adjusted for publication bias.

1. HDL cholesterol (HDL-c) < 24/40 and GDM


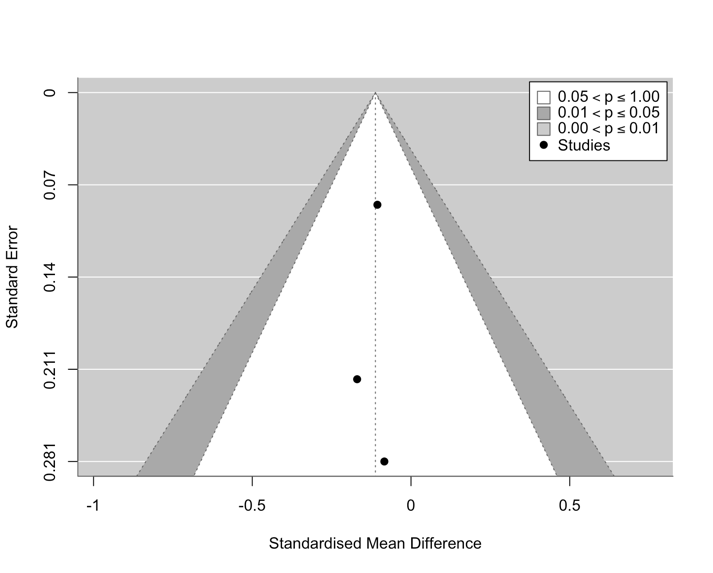


Supplementary Figure 1.13: Funnel plot showing small study effects in HDL-c <24/40-GDM association.


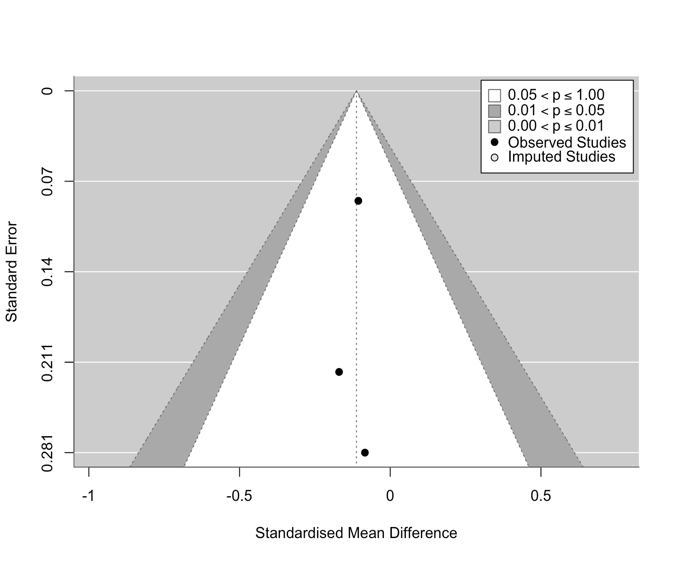


Supplementary Figure 1.14: Trim-and-Fill plot showing the summary effect of the HDL-c <24/40-GDM association adjusted for publication bias.

1. LDL cholesterol (LDL-c) < 24/40 and Gestational Diabetes Mellitus


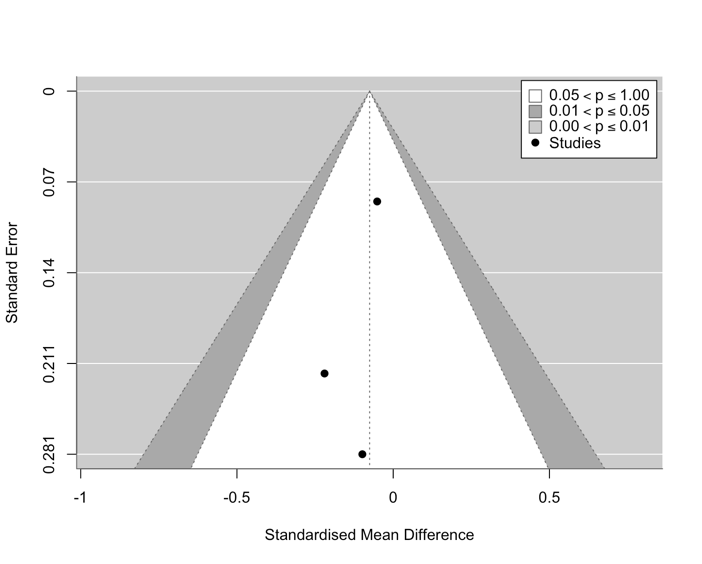


Supplementary Figure 1.15: Funnel plot showing small study effects in LDL-c <24/40-GDM association.


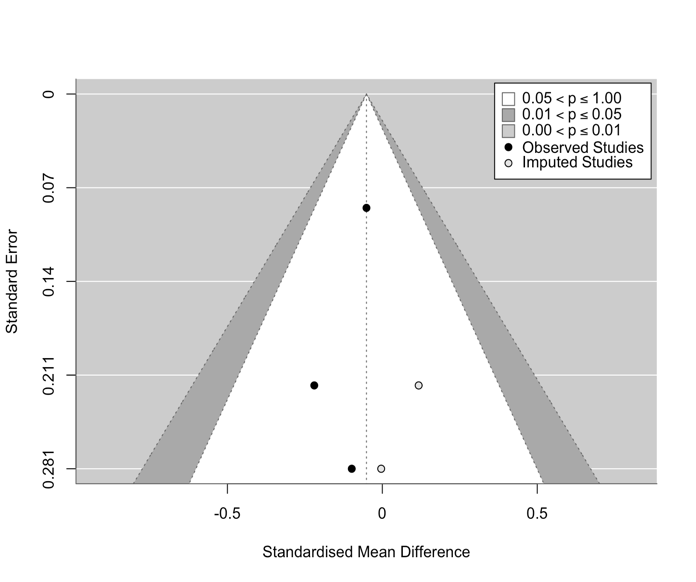


Supplementary Figure 1.16: Trim-and-Fill plot showing the summary effect of the LDL-c <24/40-GDM association adjusted for publication bias.

1. HDL-c in 2^nd^ or 3^rd^ Trimester and GDM


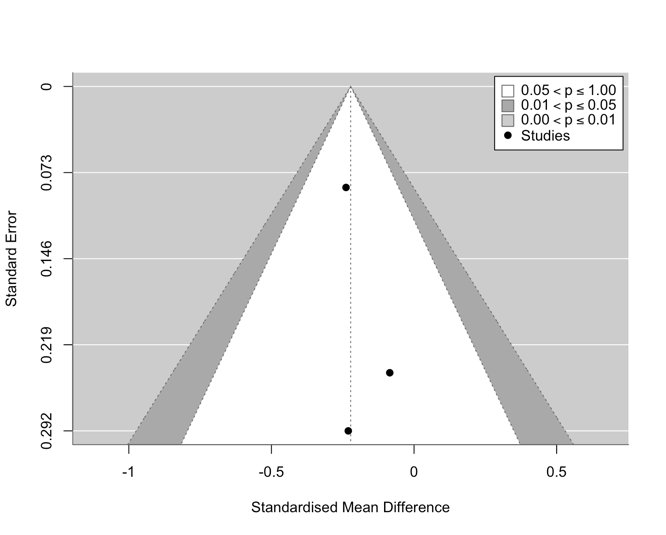


Supplementary Figure 1.17: Funnel plot showing small study effects in HDL-c-GDM association.


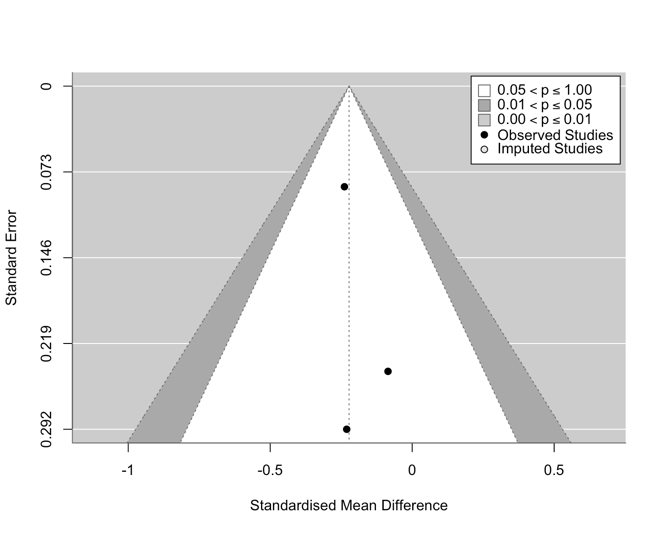


Supplementary Figure 1.18: Trim-and-Fill plot showing the summary effect of the adiponectin-preeclampsia association adjusted for publication bias.

1. LDL-c in 2^nd^ or 3^rd^ Trimester and GDM


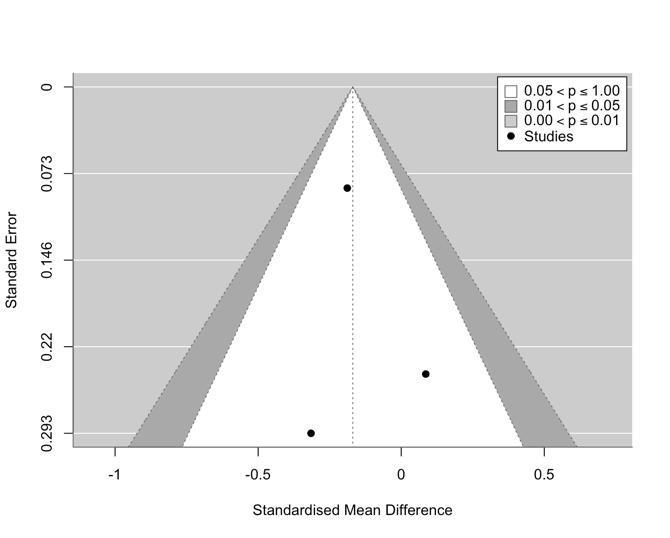


Supplementary Figure 1.19: Funnel plot showing small study effects in LDL-c-GDM association.


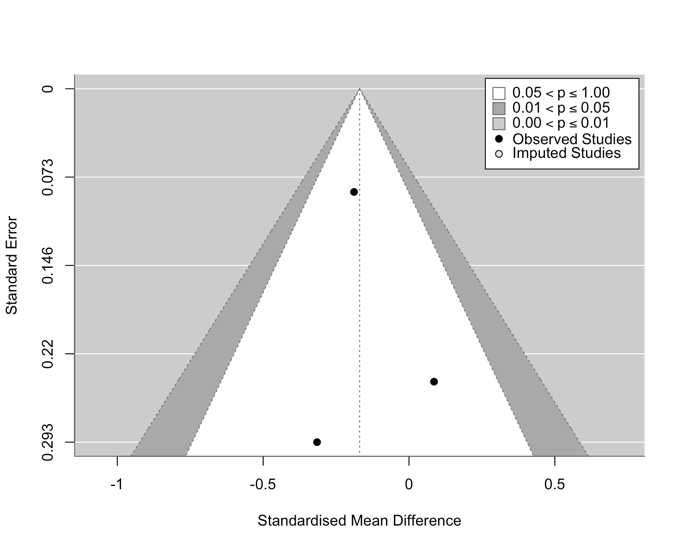


Supplementary Figure 1.20: Trim-and-Fill plot showing the summary effect of the LDL-c-GDM association adjusted for publication bias.

1. Leptin and GDM


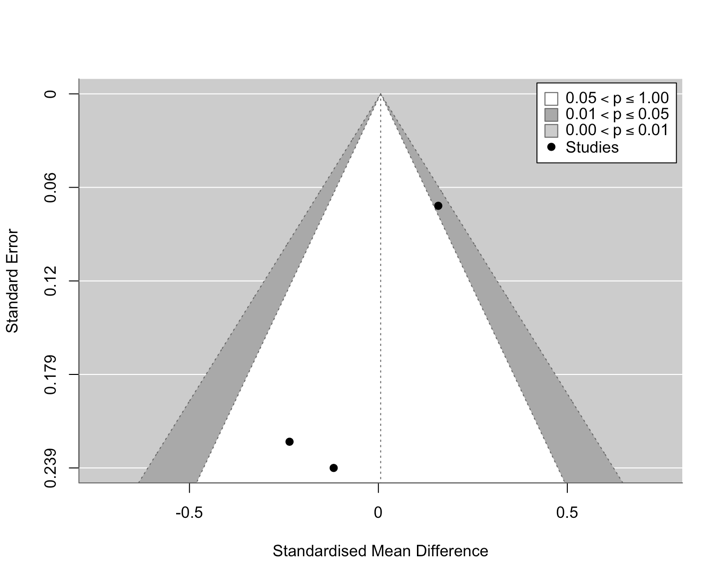


Supplementary Figure 1.21: Funnel plot showing small study effects in leptin-GDM association.


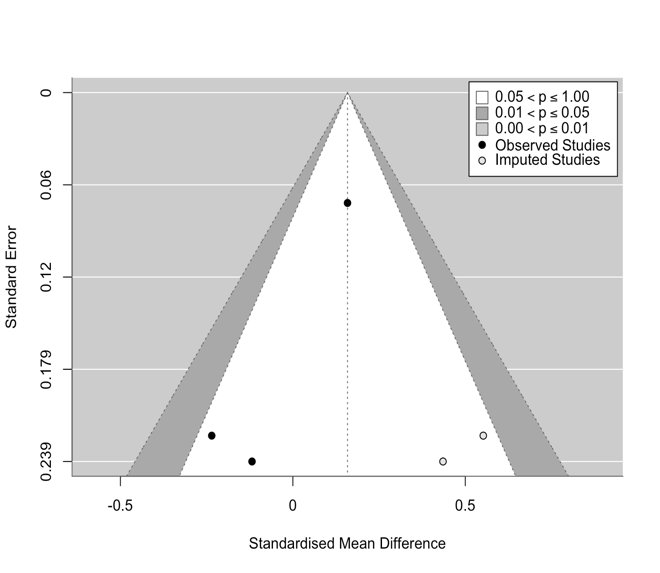


Supplementary Figure 1.22: Trim-and-Fill plot showing the summary effect of the leptin-GDM association adjusted for publication bias.

1. Alanine Aminotransferase (ALT) and Gestational Diabetes Mellitus


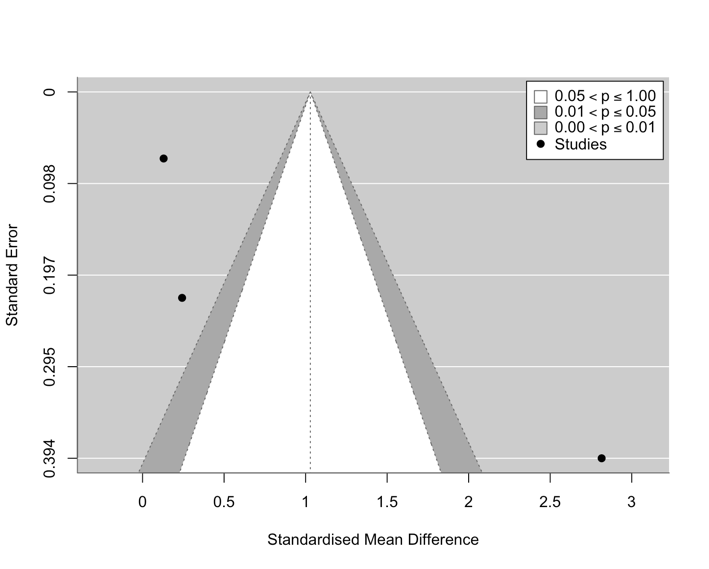


Supplementary Figure 1.23: Funnel plot showing small study effects in ALT-preeclampsia association.


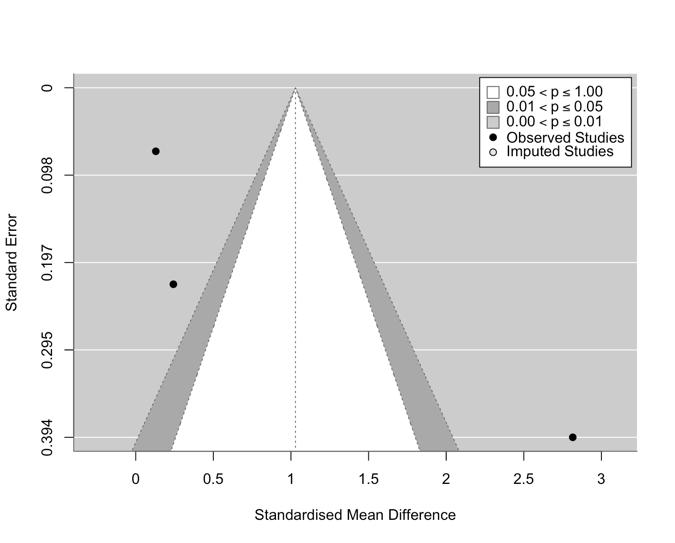


Supplementary Figure 1.24: Trim-and-Fill plot showing the summary effect of the PLGF-preeclampsia association adjusted for publication bias.

1. Adiponectin and Pre-eclampsia


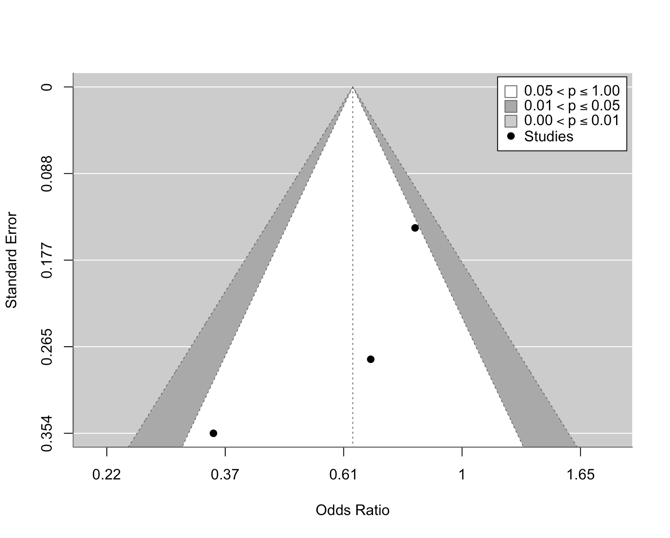


Supplementary Figure 1.25: Funnel plot showing small study effects in adiponectin-preeclampsia association.


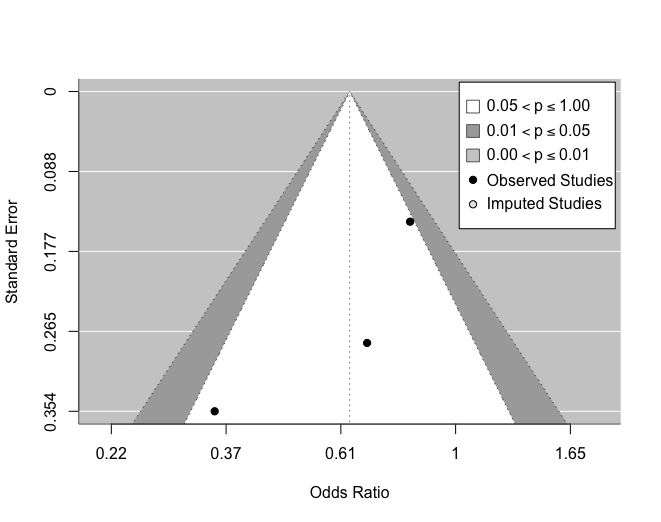


Supplementary Figure 1.26: Trim-and-Fill plot showing the summary effect of the adiponectin-preeclampsia association adjusted for publication bias.
