# Supplementary material for: Biomarkers predicting adverse pregnancy outcomes in women living with obesity: a systematic review and meta-analysis
Source: AJOG Glob Rep. 2025 Jul 22;5(3):100527. doi: 10.1016/j.xagr.2025.100527 (PMC12465041; doi:10.1016/j.xagr.2025.100527)
Supplement: Supplementary file 4 [file mmc4.docx]

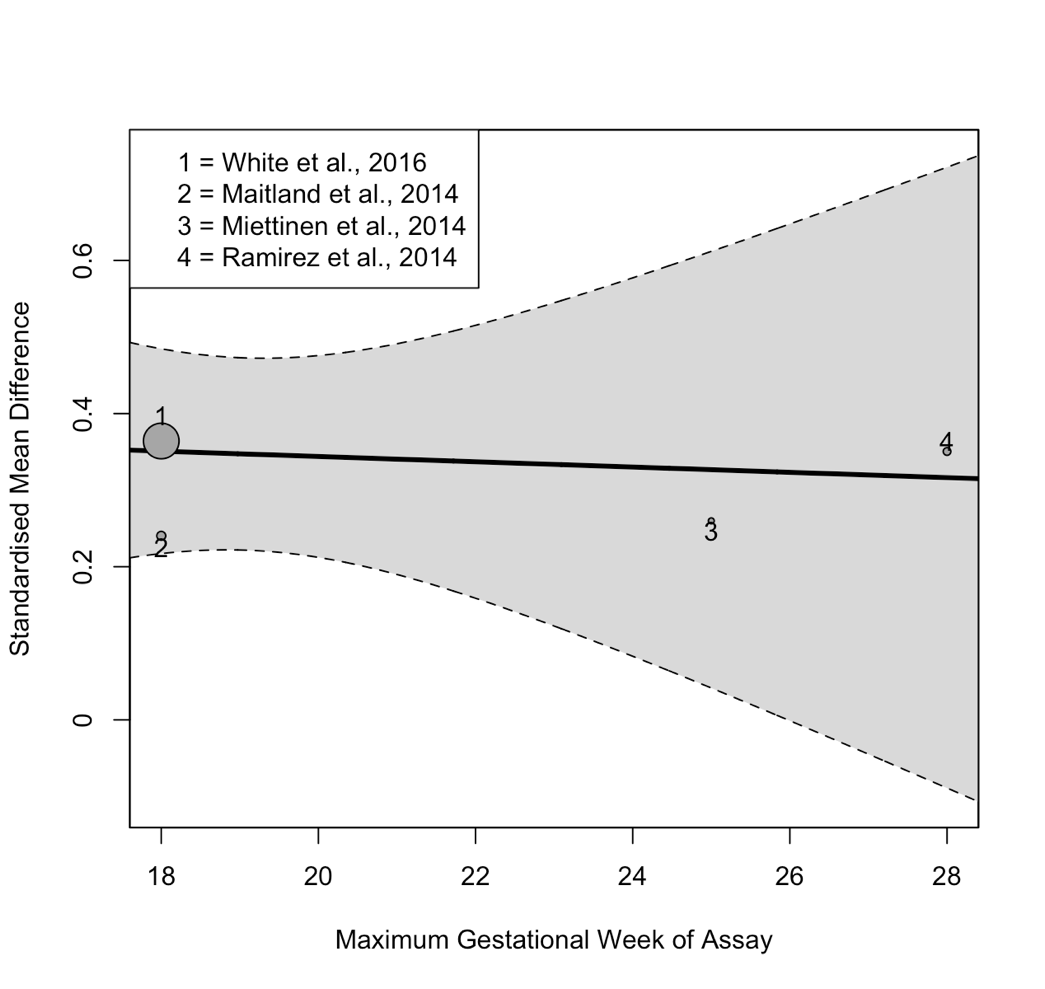


Supplementary Figure 2: Bubble plot showing study effect sizes against the maximum gestational age of insulin assay. Black line = regression line; circles = studies; dotted lines = 95% confidence interval.
