# Supplementary material for: Biomarkers predicting adverse pregnancy outcomes in women living with obesity: a systematic review and meta-analysis
Source: AJOG Glob Rep. 2025 Jul 22;5(3):100527. doi: 10.1016/j.xagr.2025.100527 (PMC12465041; doi:10.1016/j.xagr.2025.100527)
Supplement: Supplementary file 5 [file mmc5.docx]

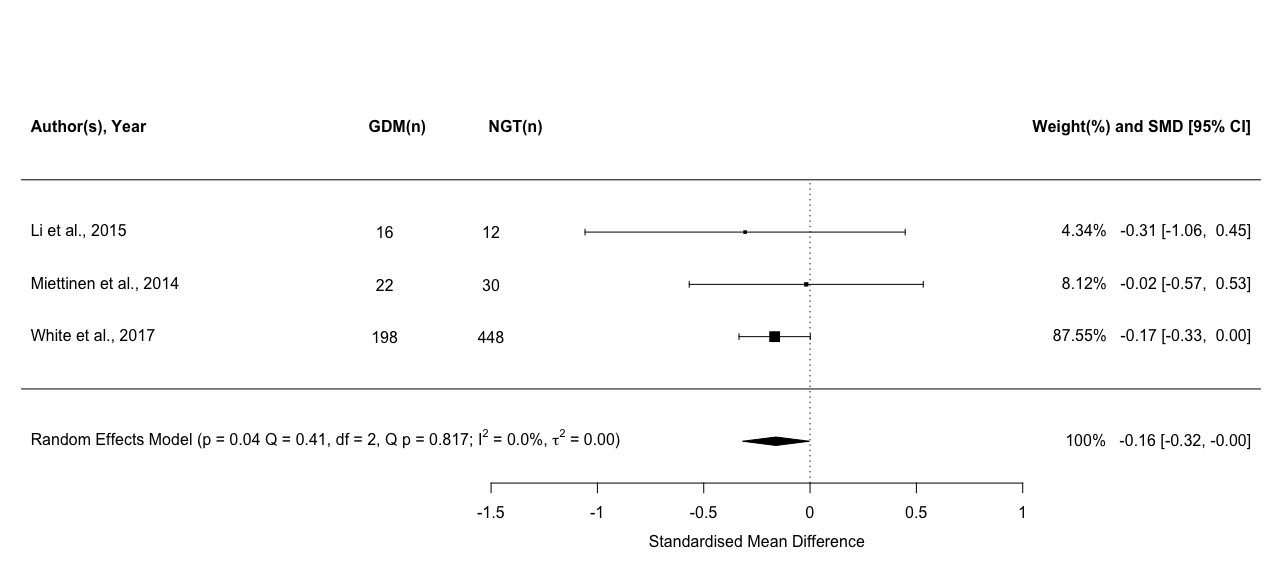


Supplementary Figure 3: Meta-analysis of total cholesterol assayed after 24 weeks’ gestation in women with a BMI ≥30kg/m² who experience GDM vs. those remaining glucose tolerant.
