# Supplementary material for: Biomarkers predicting adverse pregnancy outcomes in women living with obesity: a systematic review and meta-analysis
Source: AJOG Glob Rep. 2025 Jul 22;5(3):100527. doi: 10.1016/j.xagr.2025.100527 (PMC12465041; doi:10.1016/j.xagr.2025.100527)
Supplement: Supplementary file 7 [file mmc7.docx]

**Supplementary Table 2: Characteristics and Biomarker Associations of Papers Included in the Systematic Review**

| **Study ID** | **Title** | **Location** | **Study design** | **Dates** | **N (with obesity)** | **BMI measurement: how and when** | **Outcome(s) and Diagnostic Criteria** | **Biomarker(s) and Gestation of Assay** | **Biomarkers with Positive Association with Outcome** | **Biomarkers with Negative Association with Outcome** | **Biomarkers with No Association with Outcome** |
| --- | --- | --- | --- | --- | --- | --- | --- | --- | --- | --- | --- |
| Anelli, G.M. et al., 2018 | Mitochondrial content and hepcidin are increased in obese pregnant mothers | Italy | Prospective cohort | NR | 25 | Pre-pregnancy BMI. NR how obtained. | GDM  IADPSG | Hb/Hct: 34-36/40.  Fasting glycaemia: 24-28/40  MtDNA/hepcidin: prior to C/S | Hb and Hct ↑. No significance testing. | MtDNA and hepcidin ↓. No significance testing. | NA |
| Barquiel, B. et al., 2014 | Body weight, weight gain and hyperglycaemia are associated with hypertensive disorders of pregnancy in women with gestational diabetes | Spain | Prospective cohort | 1987-2008 | 63 | Self-report of pre-pregnancy weight. Height measured | HDP  ISSHP | Insulin/fasting glycaemia: NR.  HbA1C: monthly from GDM diagnosis until delivery. | No direct statistical analysis between obese GDM and NGT.  However, HbA1c ↑ plus obesity increases risk of HDP multiplicatively | NA | NA |
| Beernink, R.H. et al., 2022 | Early Pregnancy Serum Concentration of Secreted Frizzled-Related Protein 4, Secreted Frizzled-Related Protein 5, and Chemerin in Obese Women Who Develop Gestational Diabetes Mellitus | UK | Case-control | 2009-2014 | 150 | Recorded at 15+0 –18+6. NR how obtained. | GDM  IADPSG | sFRP4, sFRP5, chemerin  Assayed at 15+0–18+6; | NA | NA | sFRP4, sFRP5,  chemerin ↔ |
| Bogdanet, D. et al., 2022 | The Diagnostic Accuracy of Second Trimester Plasma Glycated CD59 (pGCD59) to Identify Women with Gestational Diabetes Mellitus Based on the 75 g OGTT Using the WHO Criteria: A Prospective Study of Non-Diabetic Pregnant Women in Ireland | Ireland | Case control | November 2018 – March 2020 | 124 | Measured at first antenatal visit | GDM  IADPSG | pGCD59  Assayed 24-28/40 | pGCD59 predicts GDM in obese women. ↑ predictive power in increasing classes of obesity | NA | NA |
| Bogdanet, D. et al., 2022 | The utility of first trimester plasma glycated CD59 (pGCD59) in predicting gestational diabetes mellitus: A prospective study of non-diabetic pregnant women in Ireland | Ireland | Case control | November 2018 – March 2020 | 124 | Measured at first antenatal visit | GDM  IADPSG | pGCD59  Assayed <14/40 | pGCD59 predicts GDM in obese women. ↑ predictive power in increasing classes of obesity | NA | NA |
| Borkowski, K., et al., 2020 | Mid-gestation serum lipidomic profile associations with spontaneous preterm birth are influenced by body mass index | USA | Retrospective cohort | ~2007-2011 | 34 | Self-report of pre-pregnancy BMI | sPTB  <31/40 | Targeted metabolomic profiling of oxylipins, endocannaboids, PUFA and bile acids (n = 58 oxylipins detected)  Untargeted metabolomic clusters of complex lipids and biogenic amines (n = 179):  Assayed at 15-17/40 | LOX ↑  Autooxidation metabolites ↑  18 Carbon PUFA↑  Linoleic and alpha linoleic acid ↑  LA and adrenic acid derived ethanols ↑  AA derived monoglycerides ↑  AA derived prostaglandin ↑  Triglycerides containing lipid clusters ↑  Diglyceride containing lipid clusters ↑  Phospholipid containing lipid clusters ↑ | Lysophospholipid containing lipid cluster ↓ | NA |
| Chandrasekaran, S., et al., 2020 | Adipokine Profiles in Preeclampsia | USA | Case control | Not recorded | 65 | Height and weight recorded in first trimester. | Pre-eclampsia  ACOG 2013 | Leptin, adiponectin, visfatin, resistin, TNF-Alpha, IL-17, IL-10, ILN-Gamma, IL-6, IL-1B, IL-2, IL-1Alpha, MMP2, TIMP1, TIMP2  Assayed at 28-34/40 | Visfatin ↑  Resistin ↑  IFN-Gamma ↑  IL-6↑  IL-1Beta ↑  IL-2 ↑ | NA | Leptin ↔  Adiponectin ↔ |
| Clausen, T., et al., 2005 | Maternal anthropometric and metabolic factors in the first half of pregnancy and risk of neonatal macrosomia in term pregnancies. A prospective study | Norway | Prospective cohort with nested case control | 1995-1996 | 105 | First trimester – retrieved from medical records | Macrosomia  Z score >95^th^ centile  Birthweight > 4500g | Insulin, glucose, lipids (triglycerides, total cholesterol, HDL-c, non HDL-c, high density lipoproteins)  Assayed 17-19/40 | NA | NA | Insulin ↔  Glucose↔ |
| Curry, A.E., et al., 2009 | First-trimester maternal plasma cytokine levels, pre-pregnancy body mass index, and spontaneous preterm delivery | Denmark | Case control | 1997-2002 | 170 | Self-report pre-pregnancy height and weight | sPTB  <34/40 | IL-2, IL-6, TNF-Alpha, IFN-gamma, GM-CSF  Assayed ~7-10/40 | NA | IL-2 ↓  TNF-Alpha ↓ | IL-6 ↔  IFN-Gamma ↔  GM-CSF ↔ |
| Du, C. and Kong, F, 2019 | A Prospective Study of Maternal Plasma Concentrations of Retinol-Binding Protein 4 and Risk of Gestational Diabetes Mellitus | China | Prospective cohort | July 2015 – June 2016 | 104 | Self-report height weight at first antenatal visit | GDM  Ministry of China (= IADPSG) | Retinol-Binding Protein (RBP4)  Assayed at median 6/40 (IQR 4/40–10/40) | RBP4↑ | NA | NA |
| Eid, J., et al., 2022 | The Quantose Insulin Resistance Test for Maternal Insulin Resistance: A Pilot Study | USA | Prospective cohort | December 2017 – June 2019 | 100 | Pre-pregnancy. NR how obtained. | GDM  2-step:  1-hour GTT  then  OGTT using  Carpenter and Coustan | HOMA-IR (fasting insulin, fasting glucose), Quantose IR (fasting insulin, alpha-hydroxybutyrate, oleic acid, linoleoyl-glycerophosphocholine)  Assayed 10+0 - 13+6 and 24+0 - 28+0 | In first trimester Quantose IR ↑ | NA | NA |
| Fan, H.M., et al., 2022 | Sulfated Progesterone Metabolites That Enhance Insulin Secretion via TRPM3 Are Reduced in Serum From Women With Gestational Diabetes Mellitus | UK | Case control | Cohort 3: 2012- 2018 Cohort 4: 2010-2015 | 260 | Cohort 3: NR Cohort 4: BMI at 11-13/40. NR how obtained. | GDM  Cohort 3: Modified NICE criteria (FPG ≥ 5.6 mmol/l and/or 2 hr ≥ 7.8mmol/l)  Cohort 4: If random PG ≥ 6.7 mmol/l then OGTT. Diagnosed if FPG ≥ 6 mmol/l or 2 hours ≥ 7.8mmol/l) | PM3S, PM3DiS, PM2DiS, PM5S, PM4S, PM(delta)5S  Cohort 3 assayed 26-30/40  Cohort 4 assayed 11-13/40 | NA | PM5S ↓  PM3S ↓ | PM3DiS ↔  PM2DiS ↔  PM4S ↔  PM(delta)5S ↔ |
| Fayed, M.R. et al., 2004 | Hyperhomocysteinemia is A risk marker for development of maternal pre-eclampsia | Egypt | Prospective cohort | Not recorded | 48 | 16/40. Obtained ‘by examination’. | Pre-eclampsia  No reported diagnostic criteria | Homocysteine  Assayed 16/40 | NA | NA | Homocysteine ↔ |
| Ferguson, K.K. et al., 2017 | Repeated measures of inflammation and oxidative stress biomarkers in preeclamptic and normotensive pregnancies | USA | Prospective cohort | 2006-2008 | 88 | At visit 1. NR how obtained. | Pre-eclampsia  ACOG 2013 | CRP, IL-1Beta, IL-6, IL-10, TNF-Alpha, 8-Hydroxydeoxyguanosine, 8-Isoprostane, specific gravity  Assayed at 3 median timepoints: 10/40, 18/40, 26/40, 35/40 t | 8-isoprostane ↑ | NA | NA |
| Furse, S., et al., 2022 | Altered Lipid Metabolism in Obese Women With Gestational Diabetes and Associations With Offspring Adiposity | UK | Prospective cohort | 2009-2014 | 867 | <19/40. NR how obtained. | GDM  IADPSG  LGA  ≥ 90^th^ centile on customised growth chart | 430 lipid variables  Assayed 24+2 - 30+0 | In GDM: DG(32:0) ↑, DG (34:2) ↑  TG(48:0) ↑  TG (50:1) ↑  TG (50:2) ↑  Insulin ↑  C-Peptide↑  HOMA IR ↑  TG↑ | HDL-C ↓  HOMA2b ↓ | In GDM: total triglyceride abundance ↔  Leptin ↔  Total cholesterol ↔  LDL-C ↔  In LGA DG(32:0)↔ DG(32:1)↔ DG(34:1)↔ TG(46:0)↔ TG(46:1)↔ TG(48:0)↔ TG(48:1)↔ TG(50:1)↔ TG(50:2) ↔ |
| Furse, S., et al., 2019 | Altered triglyceride and phospholipid metabolism predates the diagnosis of gestational diabetes in obese pregnancy | UK | Prospective cohort | 2009 - 2014 | 831 | <19/40. NR how obtained. | GDM  IADPSG | 565 lipid variables  Assayed 15-18/40 | CE(18:3) ↑  DG(32:0) ↑  DG(34:3) ↑  PC(38:5)↑  TG(48:0)↑  TG(50:1)↑  TG(50:2)↑  TG(51:5)↑  TG(53:4)↑  TGox(54:7)↑ | LPC(16:00)↓  LPC(18:1)↓  PC(35:02)↓  PC(40:10)↓  PC-O(40:4)↓  SM(32:1)↓  SM(41:2) ↓  SM(42:3)↓  TG(46:5)↓  TG(48:6)↓  TGox(48:2)↓  TGox(54:4)↓  TGox(56:4)↓  TGox(58:6)↓ | NA |
| Hashemipour, S., et al., 2018 | Level of maternal triglycerides is a predictor of fetal macrosomia in non-obese pregnant women with gestational diabetes mellitus | Iran | Prospective cohort | January 2015 – March 2016 | 59 | Pre-pregnancy BMI self-reported | Macrosomia  ≥ 4000g birthweight | Triglycerides - assayed 24-28/40  FPG and postprandial PG assayed ‘in the last weeks of pregnancy’ | NA | NA | Triglycerides ↔ |
| Jaaskelainen, T., et al., 2018 | Impact of obesity on angiogenic and inflammatory markers in the Finnish Genetics of Pre-eclampsia Consortium (FINNPEC) co | Finland | Case control | 2008- 2011 | 360 | Pre-pregnancy height and weight from medical records | Pre-eclampsia  Hypertension (BP ≥ 140/90)  and proteinuria (≥ 0.3g/l or ≥1+ on dipstick) >20/40 | HS-CRP, sEng, sFlt-1, PlGF  Assayed 9-15/40 and 20-42/40 | 3^rd^ trimester  s-Flt1 ↑ | NA | 1^st^ trimester  s-Flt ↔  s-PlGF ↔  s-Eng↔  sFlt-1/PlGF ↔  hs-CRP ↔  3^rd^ trimester  s-PlGF ↔  s-Eng↔  sFlt-1/PlGF ↔  hs-CRP ↔ |
| Krishnan, M., et al., 2020 | The Pacific-specific CREBRF rs373863828 allele protects against gestational diabetes mellitus in Maori and Pacific women with obesity | New Zealand | Prospective cohort | Not recorded | 112 | Measured 12+0 - 17+6 | GDM  IADPSG | CREBRF rs373863828 A (minor) allele  Triglycerides  Total cholesterol  HDL-c  LDL-c  Assayed 12+0 – 17+6 | NA | CREBRF rs373863828 A (minor) allele ↓ | Triglycerides ↔  Total cholesterol ↔  HDL-c ↔  LDL-c ↔ |
| Leon-Reyes, G. et al., 2018 | Is gestational diabetes mellitus in obese women predicted by oxidative damage in red blood cells? | Mexico | Prospective cohort | Not recorded | 26 | Pre-pregnancy. NR how obtained. | GDM  IADPSG | Lipohydroperoxides (LHP), malondialdehyde (MDA), protein carbonylation (PC), glucose, triglycerides, insulin, free fatty acids, leptin, IL-6, TNF-Alpha.  Assayed in 2nd and 3rd trimesters | LHP ↑  MDA ↑  PC ↑ | NA | NA |
| Li, X-M., et al., 2015 | Chemerin expression in Chinese pregnant women with and without gestational diabetes mellitus | China | 28 | Not recorded | 28 | Pre-pregnancy height and weight recorded | GDM  IADSPG | Triglycerides, total cholesterol, HDL, LDL, ApoA, ApoB, HOMA-IR, ALB, BUN, TNF-Alpha, chemerin  Assayed 24-48 hours before delivery (non-labouring) | TNF-Alpha ↑  HOMA-IR ↑ | BUN ↓ | Triglycerides ↔  Total cholesterol ↔  HDL ↔  LDL, ↔  ApoA ↔  ApoB ↔  ALB ↔  Chemerin ↔ |
| Logie, J., et al., 2012 | Evaluation of kisspeptin levels in obese pregnancy as a biomarker for pre-eclampsia | UK | Prospective cohort | Not recorded | 169 | Measured at booking | Pre-eclampsia  ISSHP | Kisspeptin assayed ~16/40; ~28/40, ~36/40  Fasting glucose and insulin assayed 16/40 and 28/40 | NA | Kisspeptin ↓ | Fasting insulin ↔  Fasting glucose ↔ |
| Lynch, A.M., et al., 2011 | Prepregnancy obesity and complement system activation in early pregnancy and the subsequent development of preeclampsia | USA | Prospective cohort | June 2005 – June 2008 | 116 | Pre-pregnancy weight self-reported. Height measured <20/40 | Pre-eclampsia  Hypertension (BP ≥ 140/90)  AND  proteinuria (≥ 0.3g/l or ≥1+ on dipstick) OR cerebral, epigastric, RUQ symptoms with nausea, vomiting, or haematological abnormalities  >20/40 | Bb and C3a  Assayed <20/40 (mean = 11+6) | Bb ↑  C3a ↑ | NA | NA |
| Maitland, R.A., et al., 2014 | Prediction of gestational diabetes in obese pregnant women from the UK Pregnancies Better Eating and Activity (UPBEAT) pilot trial | UK | Prospective cohort | Not recorded | 117 | BMI data collected 15+0 – 17+6. NR how obtained. | GDM  IADSPG | Fructosamine, ALT, AST, Ferritin, Adiponectin, tPA, iL-6, Leptin, Visfatin, Insulin, Cholesterol, Triglycerides, HDL, CRP, VLDL, LDL, Cholesterol:HDL, LDL:HDL  Assayed 16+0 - 18+6 | NA | Adiponectin ↓ | Fructosamine↔  ALT ↔ AST ↔  Ferritin ↔  tPA ↔  IL-6 ↔  Leptin ↔ Visfatin ↔  Insulin ↔ Cholesterol ↔  Triglycerides ↔ HDL ↔  CRP ↔  VLDL ↔  LDL ↔ Cholesterol:HDL ↔ LDL:HDL ↔ |
| Maitre, L., et al., 2014 | Urinary metabolic profiles in early pregnancy are associated with preterm birth and fetal growth restriction in the Rhea mother-child study | Greece | Case control | February 2007 – February 2008 | 53 | Pre-pregnancy BMI. NR how obtained. | PTB  <37/40  sPTB  Vaginal birth, no induction  iPTB  Induction of labour or pre-labour C/S. | Urinary N-acetyl glycoprotein  Assayed (mean, SD) 11.96(1.49) weeks | Urinary  N-acetyl glycoprotein ↑ iPTB (relationship not tested for significance) | NA | NA |
| McElwain, C. J., et al., 2024 | Defective Visceral Adipose Tissue Adaption in Gestational Diabetes Mellitus | Ireland | Case control | 2019 - 2022 | 21 | Recorded 8-12/40. | GDM  Fasting glucose > 5.1mmol/L | Triglycerides  IGF-1  PAPP-A  Leptin  Adiponectin  Adipsin  Resistin | NA | NA | Triglycerides ↔  IGF-1 ↔  PAPP-A ↔  Leptin ↔  Adiponectin ↔  Adipsin ↔  Resistin ↔ |
| Miettinen, H., et al., 2014 | Elevated serum squalene and cholesterol synthesis markers in pregnant obese women with gestational diabetes mellitus | Finland | Prospective cohort | Not recorded | 52 | Pre-pregnancy weight self-reported. Height measured. | GDM  American Diabetes Association | Total cholesterol, HDL-C, LDL-C, phospholipids, triglycerides, ALT, glucose, insulin  At 3 timepoints: 10–14/40, 22–25/40, and 34–36/40 | Glucose ↑ | NA | Total cholesterol↔  HDL-c ↔  LDL-c ↔  Phospholipids ↔  Triglycerides ↔  ALT ↔  Insulin ↔ |
| Mostafavi, E., et al., 2015 | Abdominal obesity and gestational diabetes: the interactive role of magnesium | Iran | Prospective cohort | Not recorded | 40 | Measured at 12/40 | GDM  Carpenter and Coustan | Magnesium  Assayed 24-28/40 | NA | ↓ Magnesium | NA |
| O’Malley, E.G., et al., 2020 | The use of biomarkers at the end of the second trimester to predict Gestational Diabetes Mellitus | Ireland | Prospective cohort | Not recorded | 111 | Measured. NR when. | GDM  IADPSG | Insulin, glucagon, ghrelin, c-peptide  Assayed 26-28/40 | C-Peptide ↑ | NA | Insulin ↔  Ghrelin ↔  Glucagon ↔ |
| O’Malley, E.G., et al., 2020 | Maternal obesity and dyslipidemia associated with gestational diabetes mellitus (GDM) | Ireland | Prospective cohort | 2017-2018 | 116 | Measured before study entry. | GDM  IADPSG | Triglycerides, HDL-c, Triglyceride:HDL-c ratio  Assayed 26-28/40 | Triglyceride:HDL-c ratio ↑ | HDL-c ↓ | Triglycerides ↔ |
| Quotah, O., et al., 2022 | Metabolic Profiling of Pregnant Women with Obesity: An Exploratory Study in Women at Greater Risk of Gestational Diabetes (UPBEAT) | UK | Prospective cohort | 2009 - 2014 | 231 | <19/40. NR how obtained. | GDM  IADPSG | 163 analytes  Assayed at 15-18+6 and 23+2 -30 | First assay:  glucose↑  c peptide ↑  gGT ↑  fructosamine ↑  Second assay:  C peptide ↑  Amino acids (4 of 9) ↑  Pyruvate ↑  Acetotacetate ↑  gGT↑  Fatty acid ratios (2 of 7) ↑ | First assay:  Glutamine ↓  Omega-3-TFA ↓  SHBG ↓  Adiponectin ↓  IL-6 ↓  Second assay:  Fatty acids (3 of 9) ↓  Fatty acid ratios (3 of 7) ↓  HOMA2B ↓  Adiponectin ↓  ↓ in some classes of lipids (4/14), cholesterol (8/21), cholesteryl esters (9/15), non-esterified cholesterol (8/15) and phospholipids (11/23) mainly at second assay | ↔ in most (bar 3) classes of lipids, cholesterol, cholesteryl esters, non-esterified cholesterol and phospholipids at first assay |
| Rajasingam, D. et al., 2008 | A prospective study of pregnancy outcome and biomarkers of oxidative stress in nulliparous obese women | UK and Netherlands | Prospective cohort | August 2003 – June 2005 | 385 | Measured at booking. | Pre-eclampsia  ISSHP  SGA <10^th^ centile  LGA >90^th^ centile  PTD <37/40 | Ascorbic acid, uric acid, retinol; alpha – tocopherol, gamma-tocopherol, triglycerides, total cholesterol, HDL-c, LDL-c, MDA  Assayed at 14+1-22+2/40 | Triglyceride↑ in pre-eclampsia | Ascorbic acid ↓ in SGA | Retinol ↔  Alpha-tocopherol ↔  MDA ↔  Total cholesterol ↔  HDL-c ↔  LDL-c ↔  Triglyceride ↔ in SGA and LGA |
| Ramirez, V. et al., 2014 | Adiponectin and IGFBP-1 in the development of gestational diabetes in obese mothers | USA | Prospective cohort | Not recorded | 72 | Pre-pregnancy BMI from medical records or self-reported weight. | GDM  50g GTT. If result >130mg/dl then OGTT using Carpenter and Coustan | HbA1c, insulin, leptin, TNF-alpha, Il-6, HOMA-IR, total cholesterol, triglycerides, HDL-C, VLDL, LDL-C, adiponectin, IGFBP-1, IGF-1  Assayed 24-28/40 | HbA1C ↑  HOMA-IR ↑  Triglycerides ↑  VLDL ↑ | HDL-c ↓  Adiponectin ↓  IGFBP-1 ↓ | Insulin ↔  Leptin↔  TNF-Alpha ↔  IL-6↔  Total cholesterol ↔  LDL-c ↔  IGF-1 ↔ |
| Schoonejans et al., 2024 | Serum bile acid measurements in women of European and South Asian ethnicity with or without gestational diabetes mellitus: A cohort study | UK | Prospective cohort | 2013 - 2018 | 147 | Obtained <16/40. NR how obtained. | GDM  IADPSG  or  ≥3 readings:  i. fasting glucose ≥5.6mmol/L  ii. 1-hour post-meal glucose of ≥7.8mmol/L | Insulin, HOMA-IR,  total bile acids, 12α-hydroxylated (12a-OH):non-12α-hydroxylated (non-12a-OH) bile acids ratio, cholic acid (CA), chenodeoxycholic acid (CDCA), cholic acid: chenodeoxycholic acid ratio, deoxycholic acid (DCA), lithocholic acid (LCA), primary: secondary bile acids ratio, ursodeoxycholic acid (UDCA), glycine-conjugated bile acids (G-), taurine-conjugated bile acids (T-)  Assayed 23-31/40 | Insulin ↑  HOMA-IR ↑  Total bile acids ↑  G-CDCA↑  G-CA↑  DCA↑  G-DCA↑  T-CDCA↑  CA↑  T-CA↑  CDCA↑  T-DCA↑  G-UDCA↑  UDCA↑  G-LCA↑  12α -OH ↑  Non-12α -OH ↑  12α−OH: non -12α -OH ↑  Secondary ↑  Unconjugated ↑  G-Conjugated ↑  T-Conjugated ↑  No significance testing. | CA/CDCA ↓  Primary ↓  Primary/Secondary ratio ↓  Conjugated ratio ↓  No significance testing. | LCA ↔  T-UDCA ↔  T-LCA ↔  G-Conjugated ratio ↔  T-Conjugated ratio ↔  No significance testing. |
| Shree, R., et al., 2022 | Association of fetal fraction with hypertensive disorders of pregnancy incidence and disease severity | USA | Retrospective cohort | May 2017 – December 2019 | 231 | Obtained up to 20/40. NR how obtained. | HDP  ACOG | Total cfDNA, fetal fraction  Assayed up to 20/40 | NA | NA | Total cfDNA ↔  Fetal fraction ↔ |
| Thagaard, I.N., et al., 2017 | Adiponectin and leptin as first trimester markers for gestational diabetes mellitus: A cohort study | Denmark | Retrospective cohort | June 2006 – December 2011 | 1292 | Pre-pregnancy height and weight obtained from hospital records | GDM  75g OGTT with 2-hr result >9 mmol/L | Adiponectin, leptin, adiponectin:leptin ratio  Assayed 6+0 - 14+0 | NA | Adiponectin ↓  Leptin ↓ (if BMI > 35)  Adiponectin:leptin ratio ↓ (if BMI 30-34.99) | Leptin ↔ (if BMI 30 – 34.99)  Adiponectin:leptin ratio ↔ (if BMI >35) |
| Thagaard, I.N., et al., 2019 | Leptin and Adiponectin as markers for preeclampsia in obese pregnant women, a cohort study | Denmark | Retrospective cohort | June 2006 – December 2011 | 1247 | Measured at 8-10/40 | HDP: BP ≥140/90 after 20 weeks  Pre-eclampsia  : BP ≥140/90 and 0.3g/1+ dipstick proteinuria  OR  BP ≥160/110 and subjective symptoms or eclampsia or deranged platelets, AST, ALT or creatinine.  OR  LDH >600, U/l, haptoglobin >0.3 g/l, AST/ALT >100 U/l, platelets <100 x 109/l | Adiponectin, leptin, adiponectin:leptin ratio  Assayed 8+0 - 14+6 | NA | Adiponectin ↓  Leptin ↓ (if BMI over 35) | Leptin ↔ (if BMI 30-34.99)  Adiponectin:leptin ratio ↔ |
| Tuytten, R., et al., 2023 | First-trimester preterm preeclampsia prediction with metabolite biomarkers: differential prediction according to maternal body mass index | UK | Case control | 2010 - 2015 | 312 | Recorded in first trimester. NR how obtained. | Preterm Pre-eclampsia  ACOG 2019 | PlGF, PAPP-A, Ornithine, Biliverdin, Bilirubin, 1-(1Z-octadecenyl)-2-oleoyl-sn-glycero-3-phosphocholine, decanolycarnitine, dodecanoylcarnitine, symmetric dimethylarginine, arginine-to-glutamine ratio, alanine-to-glutamine ratio, symmetric dimethylarginine-to-glutamine ratio, alanine-to-orthinine ratio, symmetric dimethylarginine-to-orthinine ratio, NG-monomethyl-L-arginine-to-orthinine ratio, glutamine-to-orthinine ratio  Assayed 11+0 - 13+6 | PlGF ↓  PAPP-A ↓ | Dodecanoylcarnitine ↑  symmetric dimethylarginine ↑  arginine-to-glutamine ratio ↑ alanine-to-glutamine ratio ↑ | Ornithine ↔  Biliverdin ↔  Bilirubin ↔  1-(1Z-octadecenyl)-2-oleoyl-sn-glycero-3-phosphocholine ↔ decanolycarnitine↔  symmetric dimethylarginine-to-glutamine ratio ↔  alanine-to-orthinine ratio ↔  symmetric dimethylarginine-to-orthinine ratio ↔  NG-monomethyl-L-arginine-to-orthinine ratio ↔ glutamine-to-orthinine ratio ↔ |
| Vieira, M.C., et al., 2018 | Gestational diabetes modifies the association between PlGF in early pregnancy and preeclampsia in women with obesity | UK | Prospective cohort | Not recorded | 824 | <19/40. NR how obtained. | Pre-eclampsia  ISSHP | HDL, triglycerides, HbA1C, adiponectin, IL-6, hs-CRP, PlGF  Assayed 15+0 - 18+6/40 | NA | PlGF ↓ | HDL ↔  Triglycerides ↔  HbA1C ↔ adiponectin ↔  IL-6 ↔  hs-CRP ↔ |
| Vieira, M.C., et al., 2017 | Clinical and biochemical factors associated with preeclampsia in women with obesity | New Zealand, Australia, UK, Ireland | Prospective cohort | Not recorded | 834 | 14-16/40. NR how obtained. | Pre-eclampsia  BP ≥140/90  and  uPCR >30mg/mmol / ≥ ++ dipstick proteinuria  or  any multisystem complication of PE (acute renal insufficiency, liver dysfunction, neurological symptoms, haematological disorders) | Adiponectin, HDL-C, ANP, BNP, Cystatin, Endoglin, PlGF  Assayed 14-16/40 | NA | PlGF ↓ | Adiponectin ↔  HDL-c ↔  ANP ↔  BNP ↔  Cystatin ↔  Endoglin ↔ |
| Wallenstein, M., et al., 2016 | Inflammatory biomarkers and spontaneous preterm birth among obese women | USA | Case control | 2009 - 2010 | 68 | Pre-pregnancy weight. NR how obtained. | sPTB  <32/40 | Adipoenctin, SCD30, SGP130, sIL-1R1, sIL-1RII, sIL-2RA, sIL-4R, sIL-6R, sRAGE, sTNFRI, sTNFRII, sVEGFR1, sVEGFR2, sVEGFR3, Leptin, SCF, MIG, MIP1-alpha, MCP3, PAI1, SFASL, ENA78, IL-1beta, IL-2, IL-4, IL-5, IP10, TGF-alpha, IL-6, IL-7, IL-8, IL-10, TGF-beta, IFN-beta, TNF-beta, IL-12P40, IL-12P70, IL-13, IL-17, PDGF-BB, NGF, IL-17F, RANTES, IFN-gamma, GMCSF, TNF-alpha, GCSF, MIP-1beta, IFN-alpha, LIF, MCP1, Eotaxin, FGF-basic, VEGF, TRAIL, GRO-alpha, IL-1alpha IL-1RA, IL-15, ICAM1, HGF, CD40L, Resistin, VCAM1, MCSF  Assayed 15-20/40 | sIL-2RA ↑  sTNFR1 ↑ | sVEGFR3 ↓ | Adipoenctin ↔  SGP130 ↔  sIL-1R1 ↔  sIL-1RII ↔  sIL-4R ↔  sIL-6R ↔  sRAGE ↔  sTNFRII ↔  sVEGFR1↔ sVEGFR2↔  Leptin↔ SCF↔ MIG↔ MIP1-alpha ↔ MCP3↔ PAI1↔ SFASL↔ ENA78↔  IL-1beta↔ IL-2↔ IL-4↔ IL-5↔ IP10↔  TGF-alpha↔  IL-6↔ IL-7↔  IL-8↔ IL-10,↔ TGF-beta↔  IFN-beta↔  TNF-beta↔  IL-12P40↔  IL-12P70↔  IL-13↔ IL-17↔ PDGF-BB↔  IL-17F↔ RANTES↔ IFN-gamma↔ GMCSF↔ TNF-alpha↔ GCSF↔ MIP-1beta↔ IFN-alpha↔ LIF↔ MCP1↔ Eotaxin↔ FGF-basic↔ VEGF↔ TRAIL↔ GRO-alpha↔  IL-1alpha ↔  IL-1RA↔  IL-15↔ ICAM1↔ HGF↔ CD40L↔ Resistin↔ VCAM1↔  MCSF↔  SCD30 ↔  NGF ↔ |
| White, S., et al., 2023 | Towards Precision Medicine in Gestational Diabetes: Pathophysiology and Glycemic Patterns in Pregnant Women With Obesity | UK | Prospective cohort | 2009-2014 | 867 | <19/40. NR how obtained. | GDM  IADPSG | 430 lipid variables and 9 biochemical markers  Assayed at OGTT (mean 27 +6/40) | All GDM subtypes:  DG(32:0)↑  DG(34:2) ↑  DG(38:6)↑  TG(48:0)↑  TG(50:1)↑  TG(50:2)↑  TG(51:1)↑  TG(51:5)↑  TG(53:1)↑  PE-O(36:5)  Fasting glycaemia subtype:  PE-O(36:5)↑  Postload hyperglycaemia subtype:  DG(34:2)↑  PC(37:3)↑  PC(39:2)↑  PE-O(38:4)↑  TG(50:1)↑  TG(50:2)↑  Leptin ↑ compared to fasting glycaemia subtype  HOMA2b ↑ compared to fasting glycaemia subtype  HOMA2s ↑ compared to fasting glycaemia subtype  Fasting and postload hyperglycaemia subtype:  DG(30:0)↑  DG(32:0)↑  DG(32:1)↑  DG(38:5)↑  DG(38:6)↑  PC(31:1)↑  PC(35:5)↑  PC(37:7)↑  TG(46:0)↑  TG(46:1)↑  TG(48:0)↑  TG(48:1)↑  TG(48:2)↑  TG(50:1)↑  TG(50:2)↑  TG(51:1)↑  TG(51:5)↑  TG(53:1)↑  Insulin ↑ compared to fasting glycaemia subtype  C-peptide ↑ compared to fasting glycaemia subtype  Triglycerides ↑ compared to fasting glycaemia subtype | Fasting and postload hyperglycaemia subtype:  CE(16:0)↓  CE(18:1)↓  CE(18:2)↓  CE(20:4)↓  Chol-lossOH↓  DG(38:0)↓  PC(35:2)↓  PC-P(40:3)↓  SM(32:1)↓  SM(33:1)↓  SM(34:1)↓  SM(34:2)↓  SM(38:1)↓  SM(40:2)↓  SM(41:2)↓  SM(42:2)↓  SM(42:3)↓  TG(40:4)↓  TG(44:5)↓  TG(46:5)↓  TG(47:5)↓  TG(48:6)↓ | NA |
| White, S., et al., 2020 | Metabolic phenotyping by treatment modality in obese women with gestational diabetes suggests diverse pathophysiology: An exploratory study | UK | Prospective cohort | 2009-2014 | 300 | <19/40. NR how obtained. | GDM  IADPSG | 89 metabolites  Assayed at 3 timepoints:  15 -18+6  23+3 - 29+6  34 - 36+0 | Diet-controlled  Timepoint 1  VLHDL↑  LHDL ↑  glucose↑  insulin↑  HDL-t ↑  LDL size ↑  HDL size ↑  Timepoint 2  VLHDL ↑  LDL size↑  HDL size↑  Timepoint 3  Glucose ↑  Insulin↑  Metformin  Timepoint 1  ELVLDL ↑  VLVLDL↑  LVLDL↑  HDL-t ↑  VLDL size ↑  SFA:TFA ↑  Isoleucine ↑  Glucose ↑  Insulin ↑  Timepoint 2  HDL-t↑  LDL size ↑  MUFA:TFA ↑  Isoleucine ↑  Glucose↑  Insulin ↑  Timepoint 3  HDL-t ↑  Alanine ↑  Insulin  Timepoint 1  Glucose ↑  Timepoint 2  ELVLDL ↑  VLVLDL↑  LVLDL↑  VLDL-t ↑  HDL-t↑  VLDL size ↑  SFA:TFA↑  Timepoint 3  Total triglycerides ↑  HDL-t ↑  Alanine ↑  Glucose ↑ | Diet controlled  Timepoint 2  VLDL- c↓  Timepoint 3:  ELVLDL↓  VLVLDL↓  LVLDL↓  MVLDL↓  SVLDL↓  VLDL-c ↓  Total triglycerides ↓  VLDL-t↓  MUFA:TFA ↓  Alanine ↓  Metformin  Timepoint 1  PUFA:TFA ↓  Timepoint 2  PUFA:TFA ↓  Insulin  Timepoint 1  LDL ↓  Timepoint 2  PUFA:TFA ↓ | All other analytes at each timepoint ↔ |
| White, S., et al., 2017 | Metabolic profiling of gestational diabetes in obese women during pregnancy | UK | Prospective cohort | 2009-2014 | 646 | <19/40. NR how obtained. | GDM  IADPSG | 163 analytes  Assayed at 2 timepoints:  15+0 - 18+6  23+2 - 30+0 | Timepoint 1  6/14 total lipid subclasses↑  1/3 particle sizes↑  2/3 apolipoproteins↑  Cholesterol in VLDL↑  Total triglycerides↑  Triglycerides in VLDL, IDL, HDL↑  Phospholipids in VLDL↑  TG:PG ratio↑  5/13 cholesterol subclasses ↑  4/14 cholesteryl ester subclasses ↑  6/14 free cholesterol subclasses ↑  9/13 triglyceride subclasses ↑  6/13 phospholipid subclasses ↑  3/9 fatty acids ↑  1/7 fatty acid ratios ↑  Glucose ↑  Pyruvate↑  Citrate ↑  4/9 amino acids ↑  ½ ketone bodies ↑  Glyc A ↑  HbA1c ↑  Insulin ↑  C-peptide ↑  Fructosamine ↑  Timepoint 2  5/14 total lipid subclasses ↑  2/3 particle sizes ↑  Cholesterol in VLDL ↑  Total triglycerides ↑  Triglycerides in VLDL, HDL↑  Phospholipids in VLDL↑  TG:PG ratio ↑  4/13 cholesterol subclasses ↑  4/14 cholesteryl ester subclasses↑  6/14 free cholesterol subclasses ↑  9/13 triglyceride subclasses↑  6/13 phospholipid subclasses↑  2/9 fatty acids↑  2/7 fatty acid ratios↑  Pyruvate↑  Citrate↑  5/9 amino acids↑  ½ ketone bodies↑  Glyc A↑  Insulin↑  C-peptide↑  Fructosamine↑  ALT↑  Ferritin↑  HOMA2-IR↑ | Timepoint 1  1/3 particle size↓  Free cholesterol in large HDL↓  Phsopholipids in VLHDL↓  3/7 fatty acid ratios↓  1/9 fatty acids ↓  SHBG↓  Adiponectin↓  Timepoint 2  VLHDL↓  LHDL↓  Apo-A1↓  Cholesterol in HDL, HDL2↓  4/13 cholesterol subclasses↓  5/14 cholesteryl ester subclasses↓  4/14 free cholesterol subclasses↓  6/13 phospholipid subclasses ↓  1/9 fatty acids ↓  4/7 fatty acid ratios↓  Adiponectin ↓  HOMA2-B↓  HOMA2-S↓ | All other analytes ↔ |
| White, S., et al., 2016 | Early Antenatal Prediction of Gestational Diabetes in Obese Women: Development of Prediction Tools for Targeted Intervention | UK | Prospective cohort | 2009-2014 | 1303 | <19/40. NR how obtained. | GDM  IADPSG | t-PA, total cholesterol, LDL-c, glucose, fructosamine, SHBG, HbA1c, insulin, C-peptide, hs-CRP, gGT, ALT, AST, triglycerides, leptin, adiponectin, ferritin, IL-6, vitamin D, human placental lactogen, HDL-c >1.5  Assayed: 15+0 - 18+6 | glucose↑  fructosamine↑  HbA1c↑  insulin↑  C-peptide↑  hs-CRP↑  gGT↑  triglycerides↑  leptin↑  50/52 lipoprotein subclasses ↑  VLDL particle size ↑  VLDL-c ↑  HDL3-c↑  Total triglycerides ↑  VLDL-t↑  HDL-t↑  Triglyceride:phosphoglyceride ratio ↑  LDL-t↑  Isoleucine↑  Leucine↑  Valine↑  Phenylalanine↑  Acetoacetate↑  Albumin↑  Pyruvate↑  Citrate↑  22:6 docosahexaenoic acid ↑  Omega 3 fatty acids ↑  Total fatty acids ↑  MUFA 16:1, 18:1 ↑  Saturated fatty acids ↑ | SHBG↓  adiponectin↓  vitamin D ↓  human placental lactogen ↓  HDL-c >1.5 ↓  1/52 lipoprotein subclasses ↓  HDL particle size↓  Glutamine ↓  Glycine ↓  PUFA:total fatty acids ratio ↓  Omega-6 fatty acids: total fatty acids ratio ↓  18:2 linoleic acid: total fatty acid ratio ↓ | t-PA ↔  total cholesterol↔ LDL-c ↔ ALT↔  AST↔, ferritin ↔ IL-6 ↔  1/52 lipoprotein subclasses ↓ |
| Wild, R.A., et al., 2023 | Highly Atherogenic Lipid Particles are Associated with Preeclampsia After Successful Fertility Treatment for Obese Women who have Unexplained Infertility | USA | Case control | Not recorded | 75 | ~ 16 weeks prior to pregnancy. NR how obtained | Pre-eclampsia  Diagnostic criteria NR | Triglycerides, HDL-c,  Triglycerides:HDL ratio; cholesterol:HDL ratio; VSLDLa, VSLDLb, VSLSLc, VSLDLd  Assayed at 3 timepoints: 16/40, 24/40, 32/40 | Timepoint 1  VSLDLa ↑  VSLDLc ↑  Timepoint 2  TG:HDL ↑  VSLDLb ↑  VSLDLc ↑  VSLDLd↑  Timepoint 3  Triglycerides ↑  Triglyceride:HDL ↑  Cholesterol:HDL ↑  VSLDLb ↑ | Timepoint 2  HDL ↓ | All others ↔ |
| Williams, Ian.M., et al., 2023 | Lipidomics Reveals Elevated Plasmalogens in Women with Obesity Who Develop Preeclampsia | USA | Case control | 2009 - 2014 | 100 | Self-reported height and weight | Pre-eclampsia  ACOG 2002 | 280 lipid species  Assayed at 3 timepoints: 6-12; 18 - 20, 34 - 36 | Timepoint 3  PE(18:2/16:1)↑  PE(18:1/22:4)↑  PE(P-18:0/20:3)↑  PE(P-16:0/20:3)↑  PE(P-18:1/20:3)↑  PE(P-18:0/20:4)↑  FFA(15:0)↑  FFA(16:1)↑  FFA(18:3)↑ | NA | Timepoint 1 ↔  Timepoint 2 ↔ |
| Xu, C., et al., 2018 | Maternal Early Pregnancy Plasma Concentration of 25-Hydroxyvitamin D and Risk of Gestational Diabetes Mellitus | China | Prospective cohort | July 2015 – June 2016 | 104 | Recorded at the first antenatal visit. NR how obtained. | GDM  Ministry of Health of China (=IADPSG) | FPG, 25(OH)D  Assayed at first antenatal visit. Gestation NR. | NA | 25(OH)D ↓ | NA |
| Yarsilikal Guleroglu, F., et al., 2022 | Clinical Value of Serum BMP-4, BMP-2, GDF-15, MMP-9, GP39 Levels in Pregnant Women with Obesity and the Related Comorbidities Diabetes Mellitus and Gestational Hypertension | Turkey | Case control | Not recorded | 101 | Pre-pregnancy BMI recorded. NR how obtained | GDM  IADPSG  GH  ISSHP | WBC, Hb, Hct, PLT, FPG, HbA1C, creatinine, AST, ALT, triglycerides, HDL-C, LDL-C, BMP-4, BMP-2, GDF-15, MMP-9, GP39  Assayed in the second or third trimester | FPG ↑ in GDM  HbA1c ↑ in GDM  BMP-4 ↑ in GDM  BMP-2 ↑ in GDM  GDF-15 ↑ in GH | NA | WBC ↔  Hb ↔  Hct↔  PLT↔  FPG↔  HbA1C ↔ creatinine ↔ AST↔  ALT↔ triglycerides↔ HDL-C↔  LDL-C↔ |

**ACOG:** American College of Obstetricians and Gynaecologists Task Force on Hypertension Guidelines; **ALB:** albumin; **ALT:** alanine transaminase; **AST:** aspartate transaminase; **BUN:** Blood Urea Nitrogen; **C/S**: caesarean section; **ELVLDL:** extremely large very low density lipoprotein; **FPG:** fasting plasma glucose; **GDM:** Gestational Diabetes Mellitus; **gGT:** gamma-glutamyl transferase; **GM-CSF**: granulocyte-macrophage colony-stimulating factor; **Hb:** haemoglobin; **Hct:** haematrocrit; **HDL-t:** triglycerides in high density lipoprotein; **HDP:** hypertensive disorders or pregnancy; **IADPSG:** International Association of Diabetes and Pregnancy Study Groups ; **iPTB:** iatrogenic pre-term birth; **ISSHP:** International Society for the Study of Hypertension in Pregnancy; **LHDL:** large HDL; **MDA:** malondialdehyde; **LVLDL:** large very low density lipoprotein; **MtDNA:**  mitochondrial DNA; **MUFA:** monounsaturated fatty acid; **MVLDL:** medium very low density lipoprotein; **NR:** not recorded; **PG:** plasma glucose; **PTB:** pre-term birth; **PUFA:** polyunsaturated fatty acid; **tPA:** tissue plasminogen activator; **RUQ:** right upper quadrant; **SFA:** saturated fatty acid; **SFRP4:** Secreted frizzled-related protein 4; **SFRP5:**  Secreted frizzled-related protein 5; **SHBG:** sex hormone binding globulin; **sPTB:** spontaneous pre-term birth; **SVLDL:** small very low density lipoprotein; **TFA:** trans fatty acid; **VLDL-t:** triglycerides in very low density lipoprotein; **VLHDL:** very large HDL; **VLVLDL:** very large very low density lipoprotein
