# Supplementary material for: Biomarkers predicting adverse pregnancy outcomes in women living with obesity: a systematic review and meta-analysis
Source: AJOG Glob Rep. 2025 Jul 22;5(3):100527. doi: 10.1016/j.xagr.2025.100527 (PMC12465041; doi:10.1016/j.xagr.2025.100527)
Supplement: Supplementary file 8 [file mmc8.docx]

**Supplementary Table 3: Sensitivity Analyses - Egger’s Test, Leave-one-out Analysis, Removal of log₂ Data, Removal of NOS < 7 Studies**

| **Outcome** | **Biomarker** | **Effect Measure** | **Significance** | **Egger’s test (p value)** | **Leave-one-out analysis** | **Sensitivity analysis removing log₂ data from OR** | **Sensitivity analysis excluding studies with NOS scores < 7** |
| --- | --- | --- | --- | --- | --- | --- | --- |
| Gestational Diabetes | Insulin | SMD | S | Pass (p = 0.53) | Pass | NA | Fail |
| Gestational Diabetes | IL6 | SMD | NS | NA (3 studies) | Pass | NA | NA (2 of 3 studies) |
| Gestational Diabetes | CRP | SMD | S | NA (2 studies) | NA (2 studies) | NA | NA (1 of 2 studies) |
| Gestational Diabetes | Chemerin | SMD | NS | NA (2 studies) | NA (2 studies) | NA | NA (1 of 2 studies) |
| Gestational Diabetes | TNF-Alpha | SMD | NS | NA (2 studies) | NA (2 studies) | NA | NA (2 of 2 studies) |
| Gestational Diabetes | Total Cholesterol ≤24 weeks | SMD | NS | Pass (p = 0.79) | Pass | NA | NA |
| Gestational Diabetes | Total Cholesterol > 24 weeks | SMD | S | NA (3 studies) | Fail on Li 2015  Fail on White 2017 | NA | Fail |
| Gestational Diabetes | HDL ≤24 weeks | SMD | NS | NA (3 studies) | Pass | NA | NA |
| Gestational Diabetes | HDL 2^nd^/3^rd^ trimester | SMD | S | NA (3 studies) | Fail on White 2017 | NA | Pass |
| Gestational Diabetes | LDL < 24 weeks | SMD | NS | NA (3 studies) | Pass | NA | NA |
| Gestational Diabetes | LDL 2^nd^/3^rd^ trimester | SMD | S | NA (3 studies) | Fail on Miettinen 2014  Fail on White 2017 | NA | Pass |
| Gestational Diabetes | Triglycerides | Difference in Medians | S | NA (2 studies) | NA (2 studies) | NA | NA (2 of 2 studies) |
| Gestational Diabetes | ALT | SMD | NS | NA (3 studies) | Fail on Miettinen 2014 | NA | Pass |
| Gestational Diabetes | Leptin | SMD | NA | NA (3 studies) | Pass | NA | NA |
| Gestational Diabetes | Adiponectin | SMD | S | NA (2 studies) | NA (2 studies) | NA | NA |
| High Birthweight | Triglycerides | OR | NS | NA (2 studies) | NA (2 studies) | NA | NA (1 of 2 studies) |
| Pre-eclampsia | Adiponectin | OR | S | NA (3 studies) | Fail on Thagaard 2019  Fail on Vieira 2017 | Pass | NA |
| Pre-eclampsia | Triglycerides | SMD | NS | NA (2 studies) | NA (2 studies) | NA | NA (1 of 2 studies) |
| Pre-eclampsia | HDL | SMD | NS | NA (2 studies) | NA (2 studies) | NA | NA (1 of 2 studies) |
| Composite | Adiponectin | SMD | S | Pass (p = 0.27) | Pass | NA | Pass |
| Composite | Leptin | SMD | NS | Pass (p = 0.89) | Pass | NA | Pass |
