# Supplementary material for: Biomarkers predicting adverse pregnancy outcomes in women living with obesity: a systematic review and meta-analysis
Source: AJOG Glob Rep. 2025 Jul 22;5(3):100527. doi: 10.1016/j.xagr.2025.100527 (PMC12465041; doi:10.1016/j.xagr.2025.100527)
Supplement: Supplementary file 9 [file mmc9.docx]

**Supplementary Table 4: GRADE Analysis**

GRADE analysis was performed on all presented meta-analyses (including those provided as supplementary materials). Publication bias was not assessed in most cases due to the paucity of meta-analysed studies. Indirectness was controlled through strict adherence to the protocol’s inclusion and exclusion criteria.

Outcome: Gestational Diabetes

| **Biomarker** | **Certainty assessment** | | | | | | | **No. of participants** | | **Effect** | **Publication Bias*** | **Certainty** |
| --- | --- | --- | --- | --- | --- | --- | --- | --- | --- | --- | --- | --- |
|  | **No. of studies** | **Study design** | **Risk of bias** | **Inconsistency** | **Indirectness** | **Imprecision** | **Other considerations** | **GDM** | **NGT** | **SMD**  **(95% CI)** |  |  |
| Insulin | 4 | Observational | Not serious | Not serious | Not serious | Serious^a^ | NA | 356 | 827 | 0.35 (0.22, 0.48) | Pass (p = 0.53) | ⨁◯◯◯  Very Low |
| Adiponectin | 2 | Observational | Not serious | Not serious | Not serious | Serious^b^ | Large magnitude of effect | 304 | 755 | -0.57 (-0.70, -0.43) | NA | ⨁⨁◯◯  Low |
| IL-6 | 3 | Observational | Not serious | Not serious | Not serious | Serious^c^ | NA | 332 | 795 | 0.09 (-0.04, 0.22) | NA | ⨁◯◯◯  Very Low |
| CRP | 2 | Observational | Not serious | Not serious | Not serious | Serious^d^ | NA | 304 | 755 | 0.18 (0.05, 0.32) | NA | ⨁◯◯◯  Very Low |
| Chemerin | 2 | Observational | Not serious | Serious^e^ | Not serious | Serious^f^ | NA | 66 | 112 | -0.13 (-0.80, 0.53) | NA | ⨁◯◯◯  Very Low |
| TNF-A | 2 | Observational | Not serious | Serious^g^ | Not serious | Serious^h^ | NA | 46 | 54 | -1.45 (-3.70, 0.79) | NA | ⨁◯◯◯  Very Low |
| Total Cholesterol <24/40 | 4 | Observational | Not serious | Not serious | Not serious | Serious^i^ | NA | 284 | 632 | -0.00 (-0.14, 0.14) | NA | ⨁◯◯◯  Very Low |
| Total Cholesterol >24/40 | 3 | Observational | Not serious | Not serious | Not serious | Serious^j^ | NA | 236 | 490 | -0.16 (-0.32, -0.00) | NA | ⨁◯◯◯  Very Low |
| Triglycerides | 2 | Observational | Serious^k^ | Not serious | Not serious | Serious^l^ | NA | 275 | 660 | **DM (95% CI)**: 17.01 (4.77, 29.26) | NA | ⨁◯◯◯  Very Low |
| HDL < 24/40 | 3 | Observational | Not serious | Not serious | Not serious | Serious^m^ | NA | 249 | 555 | -0.11 (-0.26, 0.04) | NA | ⨁◯◯◯  Very Low |
| HDL 2^nd^/3^rd^ trimesters | 3 | Observational | Not serious | Not serious | Not serious | Serious^n^ | NA | 253 | 509 | -0.22 (-0.37, -0.07) | NA | ⨁◯◯◯  Very Low |
| LDL <24/40 | 3 | Observational | Not serious | Not serious | Not serious | Serious^o^ | NA | 249 | 555 | -0.08 (-0.23, 0.07) | NA | ⨁◯◯◯  Very Low |
| LDL 2^nd^/3^rd^ trimesters | 3 | Observational | Not serious | Not serious | Not serious | Serious^p^ | NA | 253 | 509 | -0.17 (-0.32, -0.02) | NA | ⨁◯◯◯  Very Low |
| ALT | 3 | Observational | Not serious | Serious^q^ | Not serious | Serious^r^ | NA | 325 | 785 | 1.03 (-0.66, 2.72) | NA | ⨁◯◯◯  Very Low |
| Leptin | 3 | Observational | Not serious | Not Serious | Not serious | Serious^s^ | NA | 333 | 794 | 0.01 (-0.26, 0.27) | NA | ⨁◯◯◯  Very Low |

**CI**: confidence interval; **SMD**: standardised mean difference; **DM**: Difference in medians *****Egger’s test for publication bias

**Explanations**

1. 3 of 4 studies have wide 95% confidence intervals crossing the line of no effect
2. 1 of 2 studies has a wide 95% confidence interval
3. 3 of 3 studies have 95% confidence intervals crossing the line of no effect
4. 1 of 2 studies has a 95% confidence interval crossing the line of no effect.
5. High level of heterogeneity between studies (I^2^ = 62%); little overlap of confidence intervals; opposite direction of effects.
6. 2 of 2 studies have 95% confidence intervals crossing the line of no effect. 1 of 2 studies has wide confidence intervals.
7. High level of heterogeneity between studies (I^2^ = 93%); p = <0.01; no overlap of confidence intervals.
8. 1 of 2 studies has a wide 95% confidence interval crossing the line of no effect. 1 of 2 studies have wide 95% confidence intervals.
9. 4 of 4 studies have 95% confidence intervals crossing the line of no effect. 3 of 4 studies have wide 95% confidence intervals.
10. 2 of 3 studies have wide 95% confidence intervals crossing the line of no effect
11. One study had high risk of methodological bias (NOS = 4)
12. 1 of 2 studies has a wide 95% confidence interval crossing the line of no effect.
13. 2 of 3 studies have wide 95% confidence intervals. 3 of 3 studies have 95% confidence intervals crossing the line of no effect.
14. 2 of 3 studies have wide 95% confidence intervals crossing the line of no effect.
15. 2 of 3 studies have wide 95% confidence intervals. 3 of 3 studies have 95% confidence intervals crossing the line of no effect.
16. 2 of 3 studies have wide 95% confidence intervals crossing the line of no effect.
17. High level of heterogeneity between studies (I^2^ = 98.12%); p = <0.01; little overlap of confidence intervals.
18. 1 of 3 studies has a wide 95% confidence interval. 1 of 3 studies has a wide 95% confidence interval crossing the line of no effect.
19. 2 of 3 studies have wide 95% confidence intervals crossing the line of no effect.

Outcome: High Birthweight

| **Biomarker** | **Certainty assessment** | | | | | | | **No. of participants** | **Effect** | **Publication Bias*** | **Certainty** |
| --- | --- | --- | --- | --- | --- | --- | --- | --- | --- | --- | --- |
|  | **No. of studies** | **Study design** | **Risk of bias** | **Inconsistency** | **Indirectness** | **Imprecision** | **Other considerations** |  | **OR**  **(95% CI)** |  |  |
| Triglycerides | 2 | Observational | Not serious | Not serious | Not serious | Serious^a^ | NA | 915 | 1.14 (0.80, 1.64) | NA | ⨁◯◯◯  Very Low |

**CI**: confidence interval; **OR**: odds ratio; *Egger’s test for publication bias

**Explanations**

1. 1 of 2 studies has a wide 95% confidence interval crossing the line of no effect.

Outcome: Pre-eclampsia

| **Biomarker** | **Certainty assessment** | | | | | | | **No. of participants** | **Effect** | **Publication Bias*** | **Certainty** |
| --- | --- | --- | --- | --- | --- | --- | --- | --- | --- | --- | --- |
|  | **No. of studies** | **Study design** | **Risk of bias** | **Inconsistency** | **Indirectness** | **Imprecision** | **Other considerations** |  | **OR**  **(95% CI)** |  |  |
| Adiponectin | 3 | Observational | Not serious | Serious^a^ | Not serious | Serious^b^ | Large magnitude of effect | 2528 | 0.63 (0.40, 0.99) | NA | ⨁⨁◯◯  Low |
| Triglycerides | 2 | Observational | Not serious | Not serious | Not serious | Serious^c^ | NA | PE = 44  Normotensive = 131 | **SMD:** 0.23 (-0.14, 0.60) | NA | ⨁◯◯◯  Very Low |
| HDL | 2 | Observational | Not serious | Not serious | Not serious | Serious^d^ | NA | PE = 40  Normotensive = 116 | **SMD:** -0.00 (-0.37, 0.37) | NA | ⨁◯◯◯  Very Low |

**CI**: confidence interval; **OR**: odds ratio; **SMD**: standardised mean difference; *****Egger’s test for publication bias

**Explanations**

1. High level of heterogeneity between studies (I2 = 61.5%); poor overlap of confidence intervals.
2. 2 of 3 studies have wide 95% confidence intervals. 2 of 3 studies have 95% confidence intervals crossing the line of no effect.
3. 2 of 2 studies have wide 95% confidence intervals crossing the line of no effect.
4. 2 of 2 studies have wide 95% confidence intervals crossing the line of no effect.

Outcome: Composite Poor Pregnancy Outcome

| **Biomarker** | **Certainty assessment** | | | | | | | **No. of participants** | **Effect** | **Publication Bias*** | **Certainty** |
| --- | --- | --- | --- | --- | --- | --- | --- | --- | --- | --- | --- |
|  | **No. of studies** | **Study design** | **Risk of bias** | **Inconsistency** | **Indirectness** | **Imprecision** | **Other considerations** |  | **SMD (95% CI)** |  |  |
| Adiponectin | 5 | Observational | Not serious | Not serious | Not serious | Serious^a^ | Large magnitude of effect | Affected = 444 Unaffected = 1995 | -0.52 (-0.63, -0.41) | Pass (p = 0.28) | ⨁⨁◯◯  Low |
| Leptin | 5 | Observational | Not serious | Serious^b^ | Not serious | Serious^c^ | NA | Affected = 403 Unaffected = 857 | 0.05 (-0.37, 0.26) | Pass (p = 0.89) | ⨁◯◯◯  Very Low |

**CI**: confidence interval; **SMD**: standardised mean difference; *****Egger’s test for publication bias

**Explanations**

1. 3 of 5 studies have wide 95% confidence intervals. 2 of 5 studies have 95% confidence intervals crossing the line of no effect.
2. High level of heterogeneity between studies (I^2^ = 63.17%, p = 0.05); opposite direction of effects.
3. 4 of 5 studies have wide 95% confidence intervals crossing the line of no effect.
