# Supplementary material for: Biomarkers predicting adverse pregnancy outcomes in women living with obesity: a systematic review and meta-analysis
Source: AJOG Glob Rep. 2025 Jul 22;5(3):100527. doi: 10.1016/j.xagr.2025.100527 (PMC12465041; doi:10.1016/j.xagr.2025.100527)
Supplement: Supplementary file 10 [file mmc10.docx]

**Supplementary Table 5: Sensitivity Analysis Removing UPBEAT Papers From Gestational Diabetes Mellitus Meta-Analyses**

Sensitivity analysis was performed when there were ≥ 3 studies included in a meta-analysis with an outcome of gestational diabetes, and one included study used data drawn from the UPBEAT trial. Analysis is presented as the result of the meta-analysis with the UPBEAT paper included vs. the result with the UPBEAT paper excluded.

| Biomarker | Studies (n) | UPBEAT Study | Result (UPBEAT Paper Included): SMD (95% CI, p value) | Result (UPBEAT Paper Excluded):  SMD (95% CI, p value) |
| --- | --- | --- | --- | --- |
| Insulin | 4 | White, 2016 | 0.35 (0.22, 0.47, p <0.001) | 0.28 (0.01, 0.56, p = 0.04) |
| Il-6 | 3 | White, 2016 | 0.09 (-0.04, 0.22, p = 0.63) | -0.02 (-0.34, 0.30, p = 0.89) |
| Cholesterol < 24/40 | 4 | White, 2017 | -0.00 (-0.14, 0.14, p = 0.96) | 0.03 (-0.23, 0.28, p = 0.84) |
| Cholesterol > 24/40 | 3 | White, 2017 | -0.16 (-0.32, - 0.00, p = 0.04) | -0.12 (-0.56, 0.33, p = 0.60) |
| HDL < 24/40 | 3 | White, 2017 | -0.11 (-0.26, 0.04, p = 0.14) | -0.14 (-0.48, 0.02, p = 0.43) |
| HDL 2^nd^/3^rd^ trimesters | 3 | White, 2017 | -0.22 (-0.37, -0.07, p = <0.01) | -0.14 (-0.51, 0.22, p = 0.44) |
| LDL < 24/40 | 3 | White, 2017 | -0.08 (-0.23, 0.07, p = 0.32) | -0.17 (-0.51, 0.16, p = 0.31) |
| LDL 2^nd^/3^rd^ trimesters | 3 | White, 2017 | -0.17 (-0.32, -0.17, p = 0.03) | -0.08 (-0.47, 0.31, p = 0.68) |
| Leptin | 3 | White, 2016 | 0.01 (-0.26, 0.27, p = 0.96) | -0.18 (-0.50, 0.14, p = 0.27) |
| Alanine Aminotransferase | 3 | White, 2016 | 1.93 (-0.66, 2.72, p = 0.23) | 1.50 (-1.01, 4.03, p = 0.24) |

Supplementary Table 5: Sensitivity analysis removing UPBEAT papers from GDM meta-analyses
