# Supplementary material for: Biomarkers predicting adverse pregnancy outcomes in women living with obesity: a systematic review and meta-analysis
Source: AJOG Glob Rep. 2025 Jul 22;5(3):100527. doi: 10.1016/j.xagr.2025.100527 (PMC12465041; doi:10.1016/j.xagr.2025.100527)
Supplement: Supplementary file 11 [file mmc11.docx]

| **Outcome** | **Biomarker Assayed** | **Gestation** | **Standardised Mean Difference** | **95% Confidence Interval** | **Study no. (*n*)** | **I2(%)** | **P Value** |
| --- | --- | --- | --- | --- | --- | --- | --- |
| GDM | Insulin | <29/40 | 0.35 | 0.22, 0.47 | 4 | 0.00% | <0.001 |
| GDM | Chemerin | <19/40 and 24-48 hours before delivery | -0.13 | -0.80, 0.53 | 2 | 62.68% | 0.69 |
| GDM | TNF-Alpha | 24-28/40 and 24-48 hours before delivery | -1.46 | -3.70, 0.78 | 2 | 93.71% | 0.20 |
| GDM | IL-6 | <19/40 and 24-28/40 | 0.09 | -0.04, 0.22 | 3 | 0.00% | 0.09 |
| GDM | CRP | <19/40 | 0.18 | 0.05, 0.32 | 2 | 0.00% | <0.01 |
| GDM | Adiponectin | <19/40 | -0.57 | -0.70, -0.43 | 2 | 0.00% | <0.001 |
| GDM | Total cholesterol | <24/40 | -0.00 | -0.14, 0.14 | 4 | 0.00% | 0.96 |
| GDM | Total cholesterol | >24/40 | (Difference of Medians) 17.01 | 4.77, 29.26 | 2 | 0.00% | <0.01 |
| GDM | Alanine aminotransferase | <24/40 | 1.03 | -0.66, 2.72 | 3 (1110) | 98.12% | 0.23 |
| GDM | Leptin | <28/40 | 0.01 | -0.26, 0.27 | 3 (1127) | 48.40% | 0.96 |
| GDM | HDL | ≤24/40 | -0.11 | -0.26, 0.04 | 3 (804) | 0.00% | 0.14 |
| GDM | HDL | 2nd/3rd trimesters | -0.22 | -0.37, - 0.07 | 3 (766) | 0.00% | <0.01 |
| GDM | LDL | ≤24/40 | -0.08 | -0.23, 0.07 | 3 (804) | 0.00% | 0.32 |
| GDM | LDL | 2nd/3rd trimesters | -0.17 | -0.32, -0.17 | 3 (766) | 0.00% | 0.03 |
| PET | Triglycerides | <20/40 | 0.23 | -0.14, 0.60 | 2 (156) | 0.00% | 0.22 |
| PET | HDL | <20/40 | -0.00 | -0.37, 0.37 | 2(156) | 0.00% | 0.99 |
| Composite | Leptin | <34/40 | 0.05 | -0.37, 0.26 | 5 (1260) | 63.17% | 0.74 |

*Supplementary Table 6: Results of Random Effects Models Testing for Associations Between Antenatal Biomarkers and Poor Pregnancy Outcomes*
